# Stochastic block models: A comparison of variants and inference methods

**DOI:** 10.1371/journal.pone.0215296

**Published:** 2019-04-23

**Authors:** Thorben Funke, Till Becker

**Affiliations:** 1 Production Systems and Logistic Systems, BIBA - Bremer Institut für Produktion und Logistik GmbH at the University of Bremen, Bremen, Bremen, Germany; 2 Faculty of Production Engineering, University of Bremen, Bremen, Bremen, Germany; 3 Faculty of Business Studies, University of Applied Sciences Emden/Leer, Emden, Lower Saxony, Germany; University of Bath, UNITED KINGDOM

## Abstract

Finding communities in complex networks is a challenging task and one promising approach is the Stochastic Block Model (SBM). But the influences from various fields led to a diversity of variants and inference methods. Therefore, a comparison of the existing techniques and an independent analysis of their capabilities and weaknesses is needed. As a first step, we review the development of different SBM variants such as the degree-corrected SBM of Karrer and Newman or Peixoto’s hierarchical SBM. Beside stating all these variants in a uniform notation, we show the reasons for their development. Knowing the variants, we discuss a variety of approaches to infer the optimal partition like the Metropolis-Hastings algorithm. We perform our analysis based on our extension of the Girvan-Newman test and the Lancichinetti-Fortunato-Radicchi benchmark as well as a selection of some real world networks. Using these results, we give some guidance to the challenging task of selecting an inference method and SBM variant. In addition, we give a simple heuristic to determine the number of steps for the Metropolis-Hastings algorithms that lack a usual stop criterion. With our comparison, we hope to guide researches in the field of SBM and highlight the problem of existing techniques to focus future research. Finally, by making our code freely available, we want to promote a faster development, integration and exchange of new ideas.

## Introduction

The approach of modeling systems as complex networks has spread into various disciplines from sociology over physics to engineering. The most simple form of a complex network consists of nodes and edges, where nodes represent the viewed elements and edges the relationships between them. Based on this model, various analyses such as the determination of node centralities or the robustness of the complete system can be performed easily.

**Fig 1:**
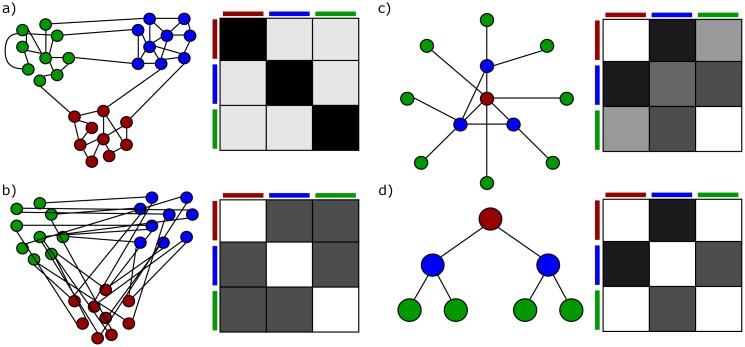
General structures and their representation as a standard SBM. The standard SBM is represented as a block matrix with the probabilities visualized in a grey-scale. a) assortative structure b) disassortative structure c) core-periphery d) hierarchy.

One aspect of particular interest is to determine elements with similar properties based on the observed and modeled relationships. Examples range from groups of friends, which attracted attention since the beginning of this research field [1], to more recent works such as grouping regions of the brain [2]. Since there are numerous applications possible, many approaches exist for the detection of these so called clusters or groups. For a more general overview of clustering methods we recommend the review of Fortunato [3]. In this publication, we focus solely on the Stochastic Block Model (SBM) because this model is unlike others based on a generative formulation. The result of a SBM inference is not only a partition, but also a description of the relationship between the inferred groups. This formulation allows, for example, the prediction of links [4–6]. Moreover, the described structure is not limited to assortative structures with more links inside the groups than between groups. Many other approaches are based on the assumption of assortative structures [7]. As we show in Fig 1, the SBM is able to create and describe a wide variety of different structures, whereas most approaches would be only capable to identify one kind. Young et al. showed algorithms that aim to find a partition of nodes that maximizes an objective function, such as minimum cuts or core-periphery, are just special cases of restricted SBMs [8].

Though, the simple formulation of the most basic SBM variant has attracted many authors to propose their own variants of the model, which often include a specific inference algorithm or strategy. But the different approaches were often compared to one basic SBM variant or even to other more limited approaches like modularity maximization. Therefore, our goal is to review the work that has already been done by other authors and compare both different SBM variants and inference algorithms independently. This comparison, in combination with the publication of our open-source code [9], is supposed to give newcomers and experts an easier application of different SBMs or the development of new approaches, be they models or algorithms.

Our comparison is based on the method of the well-known Lancichinetti-Fortunato-Radicchi (LFR) benchmark [10] and extends its set of test cases with a selection of real world networks and older test suites like the Girvan and Newman (GN) test [11]. Due to the usage of these established frameworks, we enable the comparison of our results with existing benchmarks of clustering methods [12, 13]. In contrast to these works, we investigate the separate effects of model variants and inference algorithms.

To arrange our work in the general context of community detection, we want to highlight some findings of these general comparisons as well as important general results. Peel et al. proved an approximate variant of a No Free Lunch theorem for community detection [14], which has many practical and theoretical implications. Roughly speaking, their results mean every community detection algorithm has equal performance, if the results are averaged over all possible community detection problems. As long as we restrict the subset of cases, methods can still outperform others. For a further discussion of the implication of the results, we refer to Peel et al. [14] or Ghasemian et al. [15]. Another point criticized by Hric et al. [16], that was studied more deeply by Peel et al. [14] is the differentiation between metadata of real networks and planted partitions of artificial networks. In a nutshell, the structural description given by metadata does not match well with the more topological definition of clusters used in the present community detection algorithms. Taking these results into account, we concentrate our analysis on the LFR benchmark and the GN test, which both restrict the subspace of cases as well as to avoid the problem of real network metadata with artificial networks with known planted partitions. For these kind of artificial networks some bounds are known to which the planted partition should be completely retrievable or the inferred partition should at least correlate with the planted one [17–21].

In a contrary approach, Ghasemian et al. evaluated a total of 16 community detection algorithms, of which nearly half are based on the SBM approach, on 406 real networks and evaluated the results without metadata. A major difference is however that they calculated the performance for the link prediction task and another measure, which they called link description [15]. This review brings new insights into the quality of the different approaches’ results. Yet, the authors require for each community detection method a assessment of all possible edges based on the inferred partition and they do not differentiate the effects of the proposed model and the algorithm. As we will see below, models can miss to reach their optimal results simply because their corresponding algorithm is not able to retrieve it. In the other extreme Yang et al. made a review of general clustering methods only on artificial networks, which resulted in a decision tree to support the selection of a community detection method based on number of nodes and mixing parameter [13]. The approach of Lancichinetti and Fortunato was similar. They used their proposed framework to execute some algorithms aiming to highlight the advantage of their benchmark [12]. But similar to studies like [22], the recent developments in the field of SBM were not included. Other authors concentrated their reviews on the selection of either the number of clusters or the decision between competing variants [2, 23–25]. These comparisons usually take only very few variants into account and usually come along with their own, new and most suited approach for this challenge. The most similar publication to our work was done by Zhang et al. [26]. They compared spectral clustering to expectation maximization with naive mean field and belief propagation as inference algorithms for the basic SBM. Additionally, Young et al. differentiated between objective function and maximizer, i.e. the algorithm used to maximize the objective function [8]. Though, they focused on constructing a hierarchy of objective functions of SBM and other approaches.

So comparisons of clustering detection methods are available and concentrate on different aspects with various measures and test networks. But a joint analysis and comparison of SBM variants and inference algorithms is still missing. Therefore, we try to study the effects of different choices on the results of the community detection problem.

Before presenting the SBM variants in detail, we give an overview of the development in this field and provide some additional rationale. A more exhaustive introduction into the topic of SBM can be found in [27], where Peixoto explains his microcanonical formulation from the most basic variant of SBM up to his hierarchical version. Another noteworthy overview from Abbe [28] focuses more on recovery thresholds and the theoretical properties of different approaches. The first SBM was developed in social sciences to study social networks and combine the benefits of block models like [29] and stochastic models [1]. The aim was to understand the principles behind the formation of ties between individuals. The authors of this first model assumed that the partition of the individuals is given a priori via additional attributes. To drop this assumption, Wasserman and Anderson proposed different methods to infer an optimal partition [30].

After this early development, the importance of networks in a variety of research fields from biology via physics to engineering emerged [31]. Once more community structures were observed and methods were needed to detect and describe these structural information. Together with a vast progress in available computational power, this led to the development and application of different new and old approaches including the SBM [3]. With the facilitation of the execution and processing of larger graphs, several disadvantages of the first approaches were detected and fixed by extending the SBM.

One of the most prominent enhancement was provided by Karrer and Newman, who offered a solution for the application of the SBM to complex networks with a broader degree distribution [32]. This made the SBM applicable to many real world networks, which, like the research of small-world and scale-free networks has shown, are often characterized by broad degree distributions [33]. Additionally, this work created new momentum for the research of SBM.

Peixoto, another noteworthy author, created and developed his microcanonical view on the SBM, where, roughly speaking, edge probabilities are replaced by a fixed amount of edges [2, 27, 34–39]. His work covers most of the present SBM variants and is available in form of the graph-tool module, a C++ based python package.

The majority of publications presenting new SBM variants follow a similar pattern: the model is applied to real networks to show the benefits of the specific extension. In some cases like the extension of SBM to weighted graphs, the extension is motivated by a certain set of real networks [39, 40]. Other works concentrate on the inferred community structure and its interpretation for a specific real world network, such as financial networks [41, 42], gene networks [43–45] and others [46–49]. Another field of application of the inferred structural information is the prediction of unobserved or missing edges or nodes [4, 5, 50, 51]. Beside the research with a focus on applications or new models, other authors aim to prove information theoretic theorems like detection thresholds for the planted partition [17–21]. For the planted partition model with its simplified group structure, Newman has shown the equivalence between maximizing the likelihood of SBM and maximizing a generalized modularity function, another widely used heuristic method [7]. Keeping this big picture in mind will help to understand and sort the following SBM variants.

## Variants of SBM

At first we want to take a look at the different variants of SBM in order to cover the major approaches used for application. We divide the variants into the well-established classic models and more recent extensions. We explicitly omit approaches with a focus in the information theoretic field.

Each variant section is structured into a motivation for the development of the specific variant, a general description, which highlights the advantages of this variant with some examples, and theory with the likelihood functions. The models will be stated for undirected networks with self-loops. The directed cases, if available, are included in our implementation as well.

### Classic models

The regular SBM and the degree-corrected SBM of Karrer and Newman are the most used variants of SBM [32]. Both models share the fact that they require the knowledge about the actual number of clusters **K** in the network. For simplicity, we initially assume, that this number is given a priori, and in the last section we describe means to retrieve this number from the data.

To allow a clear distinction of any SBM variant and the SBM with no addition, we call the model described in the next section the standard SBM.

#### Standard SBM

The standard SBM originates in social sciences and was developed to describe group structures in friendship networks [1, 30]. As a combination of the strict block model with a stochastic element, it was able to deal with imperfect group structures and noise of real world networks. The standard SBM does not only determine the likelihood of a specific group structure belonging to a certain network. The model is based on a generative model, which enables the user to generate other network instances from a given structure or allows the prediction of missing edges [4–6, 50, 51]. Moreover, the SBM is capable to describe any kind of group structure. Olhede and Wolfe developed network histogram based on SBM as universal representation of interactions in networks [52]. An overview of selected structures and their representation as a standard SBM is displayed in Fig 1.

The basic idea of the standard SBM is that the neighborhood relations of each node only depend on the probabilities given by the group memberships. Roughly speaking, the nodes are clustered in a way so that the neighbors of nodes in a group have a similar neighbor pattern as well. This idea becomes clearer if we first take a look at the generative model included in the SBM and then describe the reverse process of SBM inference from a given network.

#### Generative model

To explain the generative model of the SBM, we assume all model parameters are given. For the standard SBM in its most simple formulation the model parameters are the group structure **b** = *b*_1_, …, *b*_*K*_, which assigns each node to a single group *b*_*r*_, and the edge probabilities *ω*. To create one realization of these parameters, for each node pairing their respective group assignment and the corresponding edge probability is retrieved. Then, the edge probability is evaluated and depending on the result edges are added to the network. For example an edge between a node *i* in group *r* and a node *j* in group *s* is created with probability

$$\begin{array}{c} P\left(i\to j\right)=P\left(r\to s\right)=\omega_{rs}. \end{array}$$

The standard SBM is a kind of two step Erdős-Rényi model, where first the nodes are assigned to groups and then the edges between two groups as well as inside the group are created in a Erdős-Rényi random fashion with the respective probability given by the edge probability matrix.

With the knowledge of the generative model the relationship between the edge probability matrices and the network realizations in Fig 1 are clearer. Yet, solely based on the given parameter the generation process can create very different networks based on the underlying random process. Similar to the Erdős-Rényi model, if all entries of the edge probability matrix are in the open interval (0, 1), then all networks with the given numbers of nodes can be created. But, given specific parameters, the likelihood of that a network was generated based on these parameters can be easily calculated. For the later inference task it is more important that the likelihood of the parameters given a network can be calculated, too. Fixing either a network or a set of parameters results in a probability distribution of the other space. This duality between networks and sets of parameters is the starting point of the inference task.

#### Theory

The main inference task of all SBM variants is either to sample from the probability space of parameters or to approximate the optimal set of parameters. In both cases, the likelihood of a set of parameters is needed.

Before we start stating and explaining any likelihood functions, we adjust the above stated generative model. In the description of the generative process, a Bernoulli distributions is used to decide whether an edge is created or not. Some of the early works are based on these model [1, 53]. For completeness, we state one of the likelihoods based on the Bernoulli distribution, before turning to the Poisson distribution, which is easier to handle.

First we need some basic notation. Let *G* = (*V*, *E*) be a graph with |*V*| = *N* nodes and |*E*| = *M* edges. The aim is to find a partition **b** = *b*_1_, …, *b*_*K*_ of the nodes into *K* groups, i.e. each node is in exactly one of the *b*_*r*_ or in other words ∪_*r*_*b*_*r*_ = *V* and *b*_*r*_ ∩ *b*_*s*_ = ∅ for all *r*, *s* ∈ {1, …, *K*}. For the formulation of Snijders et al. [53], we need in addition a stochastic vector $\tilde{b}=\{{\tilde{b}}_{1},\ldots ,{\tilde{b}}_{K}\}$, where ${\tilde{b}}_{r}$ is the likelihood of a node belonging to group *r*, and the likelihoods for edges between the groups *ω* = (*ω*_*rs*_)_*r*,*s* = 1*K*_. With this the likelihood is (see Eq (2) in [53])

$$\begin{array}{c} P\left(G,b|\omega ,\tilde{b}\right)={\tilde{b}}_{1}^{n_{1}}\cdots {\tilde{b}}_{K}^{n_{K}}\prod_{r\leq s}\omega_{rs}^{e_{rs}}{\left(1-\omega_{rs}\right)}^{n_{rs}-e_{rs}} \end{array}$$ (1)

where *n*_*r*_ is the number of nodes in group *r*, i.e. *n*_*r*_ = |*b*_*r*_|, and

$$\begin{array}{c} n_{rs}=\{\begin{array}{c} n_{r}n_{s}\;\text{if}\;r\neq s\\ \frac{n_{r}\left(n_{r}+1\right)}{2}\;\text{if}\;r=s \end{array} \end{array}$$

is the number of possible edges between group *r* and group *s*. Further, we denote

ers=11+δrs∑inodeofgroupr,jnodeofgroupsAij

as the number of edges between group *r* and *s* with *A* = (*A*_*ij*_) as the adjacency matrix of *G* and the Kronecker delta *δ*_*rs*_ = 1 for *r* = *s* and 0 for *r* ≠ *s*. With this formulation, one can directly see the general structure of SBM. The likelihood in Eq (1) splits into a first part representing the likelihood of node partition and the product over all pairs of groups and a second part corresponding to the likelihood of all edges. The last part of this equation not only takes existing edges into account, but also non existing edges.

For calculations, a Poisson distribution is easier to handle and because this is the basis of all extensions, we focus on this formulation. With this, a created network can now have multiple edges between two nodes. This fact is usually neglected, because the edge probabilities are often small or, like in other random models, one can simply replace any multiple edge with a single edge. With the assumption of a Poisson distribution, Karrer and Newman deduced

$$\begin{array}{c} L_{t}^{\text{KN}}\left(G|b\right)=\frac{1}{2}\sum_{rs}e_{rs}\;\text{log}\;(\frac{e_{rs}}{n_{r}n_{s}}), \end{array}$$ (2)

which they called the unnormalized log likelihood for the group assignment **b**(see Eq (6) in [32]). In contrast to the original formulation of Eq (2), we include the multiplicative constant of ½, because this changes the place of the maximum during model selection. To retrieve Eq (2), the authors used a maximum likelihood estimation for the edge probabilities *ω* and neglected all terms which do not depend on the parameters of the SBM. In this formulation **b** is a partition of the nodes into groups, i.e. **b** = {*b*_1_, …, *b*_*K*_} is a set of disjoint subsets of the node set with ∪_*r*_*b*_*r*_ = *V*.

Another formulation of the SBM is more driven by combinatorics. It replaces the evaluation of a probability distribution for each edge with the distribution of a certain amount of edges between the nodes of each pair of groups. This is similar to the two possible formulations of the Erdős-Rényi model. The first one is like the variants described above and regard an Erdős-Rényi graph as *G*(*N*, *p*), where *p* is the edge probability with *p* ∈ (0, 1), and each possible network has a specific probability depending on *p*. The alternative formulation exchanges the edge probability *p* with a fixed number of edges *M* and thus regard as Erdős-Rényi graph as *G*(*N*, *M*), where an instance is uniformly chosen from all graphs with exactly *N* nodes and *M* edges. These two formulations are also available for most SBM parameters.

An author who focuses on the latter is Peixoto, who calls this formulation of the SBM the microcanonical version of SBM. In [34] he deduced for the standard SBM

$$\begin{array}{c} L_{t}^{\text{P}}=-\frac{1}{2}\sum_{rs}n_{r}n_{s}H(\frac{e_{rs}}{n_{r}n_{s}}), \end{array}$$ (3)

with the binary entropy function *H*(*x*) = −*x* log(*x*) − (1 − *x*) log(1 − *x*) and proposed for sparse graphs the approximation

$$\begin{array}{c} L_{t,\text{sparse}}^{\text{P}}≃-M+\frac{1}{2}\sum_{rs}e_{rs}\;\text{log}\;(\frac{e_{rs}}{n_{r}n_{s}}), \end{array}$$ (4)

which has the same optima as Eq (2). In Eqs (3), (4 and (6) we assume, that each graph in the ensemble has the same probability [34], and give the likelihoods of the entropies, which were originally stated by Peixoto.

#### Degree-corrected SBM

Once we have established the standard SBM, we can concentrate on the first expansion, which is so widespread that we consider it one of the classic variants. The standard SBM has the advantage of no limitation in the kind of inferred community structure. But the resulting group structure is limited in the variety of node degrees within each group [32]. Since many real world networks possess broad degree distributions like the scale free graphs, this fact hinders the application. To overcome this restriction, Karrer and Newman have developed the degree-corrected SBM [32].

In a recent publication, Newman proved the equivalence of a restricted version of degree-corrected SBM inference and modularity optimization, that is another widely used method in community detection [7]. The maximum likelihood method of the degree-corrected planted partition model, a restricted variant of the degree-corrected SBM, is equivalent to optimization of a generalized modularity.

**Fig 2:**
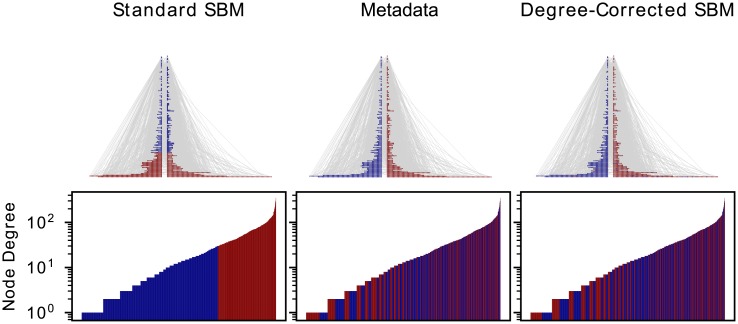
Visualization of a complex network with three partitions, the result of standard SBM and degree-corrected SBM as well as the metadata. The networks are drawn by ordering the nodes by degree with the highest at the top and splitting the nodes according to the known metadata into two sets. The color of each node represents the membership to one of the two groups given by the partition of the respective column. Below the network graphics, the degree distribution is colored according to its partitions. Each node represents a bar with the height of its degree and the color of its group.

The idea behind the degree-corrected SBM is that a new parameter *θ* = {*θ*_1_, …, *θ*_*N*_} is introduced, which controls the expected degree of each node. Before we take a look at how this parameter is included in the generative model or the resulting likelihood function, we want to highlight the advantages with some examples. In Fig 2 we can see a typical example of a local optimum of the standard SBM, where the nodes with similar degree are grouped into the same block. The results of the degree-corrected SBM show a much broader degree distribution inside inferred blocks.

#### Theory

Knowing the improvement introduced by the degree correction, the new parameter *θ* needs to be included in the model. In the standard SBM the likelihood of an edge between two nodes *i* and *j* of two distinct groups *r* and *s* is given by

$$\begin{array}{c} P\left(i\to j\right)=P\left(r\to s\right)=\omega_{rs}. \end{array}$$

The degree-corrected model allows heterogeneity inside each group and the likelihood to observe at least one edge between the same nodes is in the degree-corrected case

$$\begin{array}{c} P\left(i\to j\right)=1-\text{exp}\left(-\theta_{i}\theta_{j}\omega_{rs}\right)\;\text{for}\;i\in b_{r},j\in b_{s}\;\text{and}\;r\neq s. \end{array}$$

The likelihood function formulated by Karrer and Newman is

$$\begin{array}{c} L_{c}^{\text{KN}}=\sum_{rs}e_{rs}\;\text{log}\;(\frac{e_{rs}}{e_{r}e_{s}}), \end{array}$$ (5)

where *e*_*r*_ = ∑_*s*_ *e*_*rs*_ the total number of edges with nodes of block *r* (see Eq (23) in [32]). The microcanonical variant of the degree-corrected SBM is approximately according to Peixoto (see Eq (40) in [34])

$$\begin{array}{c} L_{c}^{\text{P}}≃M+\sum_{k}N_{k}\;\text{log}\left(k!\right)+\frac{1}{2}\sum_{rs}e_{rs}\;\text{log}\;(\frac{e_{rs}}{e_{r}e_{s}}), \end{array}$$ (6)

where *N*_*k*_ is the number of nodes with degree *k* and is beside a constant and multiplicative factor identical to (5) derived by Karrer and Newman. These differences do not change the optima for fixed number of groups, but influence the optima of the following model selection methods.

#### Selecting the number of groups

A challenge both of these classic variants of SBM share, is the requirement of the a priori knowledge about the actual number of groups. Simply minimizing one of above likelihood functions (2–6) would result in assigning each node to a different block. Different approaches to solve this problem are available and most approaches split the inference process into two steps. They first retrieve the optimal community structure for different numbers of groups and then penalize the calculated likelihood values depending on the used number of groups or more generally the number of parameters used by the model. We will present a selection of available methods and will later test the techniques in the analysis section. If not stated, all penalty functions will be added to the original likelihood and the new extreme is the optimal solution.

One example for such an approach is the minimum description length (MDL) described by [35, 37]. The penalty function of Peixoto (see Eq (9-10) in [37]) is

MDLt,Peixoto=log((KN))+logN!−∑rlognr!+log((K(K+1)2M))

for the standard SBM and

$$\begin{array}{c} MDL_{c,\text{Peixoto}}=MDL_{t,\text{Peixoto}}-\sum_{r}n_{r}\sum_{i}p_{i}^{r}\;\text{log}\;p_{i}^{r} \end{array}$$

for the degree-corrected SBM. The degree distribution of block *r* is notated with $\left(p_{i}^{r}\right)$ and $\left(\left(\begin{array}{c} n\\ k \end{array}\right)\right)=\left(\begin{array}{c} n+k-1\\ k \end{array}\right)$ is the multiset coefficient, which can be calculated using the binomial coefficient.

Another well known criterion is the Akaike information criterion (AIC; [54]), which is in general

$$\begin{array}{cc} AIC= & \;-2\;\text{log}(\text{maximum}\;\text{likelihood})\\ & +2(\text{number}\;\text{of}\;\text{independently}\;\text{adjusted}\;\text{parameters}\;\text{within}\;\text{the}\;\text{model}). \end{array}$$

The number of independently adjusted parameters is unclear for the SBM and its variants, like discussed in [24]. For the standard SBM we choose the sum *K*(*K* + 1)/2 for the edge probability matrix *ω* and *K* for the node partition as value for the number of independently adjusted parameters. For the degree-corrected model we add *K* parameters for the degree distribution inside each group, i.e. we assume the node degrees are separately sampled from a distribution for each group before the creation of edges. Another choice would be adding *N* free parameters one for the degree of each node, which would be negligible for the selection between two competing results. This criterion is widely applied in statistics, but lacks some theoretical requirements and justification for the case of SBM. Especially for sparse graphs the required precondition, that the likelihood becomes asymptotically normal in the limit of enough data, is not true [23]. As we will see in the experiments, the AIC has the tendency of overfitting, i.e. selecting too many groups. Still some authors use it together with the Bayesian information criterion (BIC; [48]).

The last tested approach is from the Bayesian context, the BIC, and following Yan we have

$$\begin{array}{c} BIC=-2\;\text{log}\;\text{likelihood}+\frac{K(K+1)}{2}\text{log}\left(N^{3}\right) \end{array}$$

for the standard SBM and

$$\begin{array}{c} BIC=-2\;\text{log}\;\text{likelihood}+\frac{K(K+1)}{2}\text{log}\left(N^{3}\right)+2\;\text{log}\left(N\right) \end{array}$$

for the degree-corrected SBM [55].

Further criteria used together with SBM variants are the integrated complete likelihood (ICL) by Daudin et al. [56], node degree gaps [57], variational Bayesian approaches [58], spectral based approaches [59], cross validation [24, 60] and ensemble methods [61].

Beside the selection criterion, an efficient way to test different values is needed. Under the assumption that the total of selection criterion and likelihood is an unimodal function, i.e. has a single extreme, we can apply search methods like golden-section search or Fibonacci search to reduce the number of tested values. With this the classical SBM variants are completely formulated and just need an inference method to be applied to any network.

### Extensions

With the knowledge of the SBM basics, we want to take a look at further extensions, which deal with a wide range of challenges like weighted graphs or the detection of smaller groups.

#### Models including number of groups

The inference process described above is inefficient in the sense that the second step of selecting the correct number of blocks multiply the complexity with a factor of at least *N* log *N*. To speed up the detection process and include the selection of the number of groups into the model, some authors refined the SBM and deduced complete formulas. The natural question, whether the following approaches performs better in accuracy and/or calculation time, we will answer in the comparison section.

One of the first authors, who achieved this goal, were Côme and Latouche [62] with the exact integrated complete likelihood (ICL)

$$\begin{array}{c} ICL_{ex}\left(b,K\right)=\sum_{r\leq s}^{K}\text{log}\frac{\Gamma \left(\eta_{rs}^{0}+\zeta_{rs}^{0}\right)\Gamma \left(\eta_{rs}\right)\Gamma \left(\zeta_{rs}\right)}{\Gamma \left(\eta_{rs}+\zeta_{rs}\right)\Gamma \left(\eta_{rs}^{0}\right)\Gamma \left(\zeta_{rs}^{0}\right)}+\text{log}\frac{\Gamma \left(\sum_{r=1}^{K}n_{r}^{0}\right)\prod_{r=1}^{K}\Gamma \left(n_{r}+n_{r}^{0}\right)}{\Gamma \left(\sum_{r=1}^{K}n_{r}+n_{r}^{0}\right)\prod_{r=1}^{K}\Gamma \left(n_{r}^{0}\right)}, \end{array}$$ (7)

where Γ is the gamma function and let

$$\begin{array}{cc} \eta_{rs} & =\eta_{rs}^{0}+e_{rs},\\ \zeta_{rs} & =\zeta_{rs}^{0}+\{\begin{array}{c} n_{r}n_{s}-e_{rs},\;\;\text{for}\;r\neq s\\ n_{r}\left(n_{r}+1\right)/2,\;\;\text{else}\; \end{array} \end{array}$$

be the pseudo counters for the number of existing and non existing edges between the group *r* and *s*. The constants $n_{k}^{0}$, $\eta_{rs}^{0}$ and $\zeta_{rs}^{0}$ are all set to ½ or 1 for a non informative Jeffrey prior or a uniform distribution. Eq (7) is the likelihood of a standard SBM including the number of groups and similar to (1) it takes with *η*_*rs*_ and *ζ*_*rs*_ the existing and non existing edges into account.

Newman and Reinert [63] obtained a closed expression for the degree-corrected SBM in

$$\begin{array}{c} P\left(K,b|G\right)=\frac{P(K)P(b|K)P(G|b)}{P(G)}, \end{array}$$ (8)

where

$$\begin{array}{c} P(K)=\frac{1}{N} \end{array}$$ (9)

$$\begin{array}{c} P(b|K)=\frac{(K-1)!}{(N+K-1)!}\prod_{r}n_{r}! \end{array}$$ (10)

and with $p=\frac{2M}{N^{2}}$

$$\begin{array}{c} P_{\text{standard}\;\text{SBM}}\left(G|b\right)=\prod_{r}\frac{e_{rr}!}{{\left(\frac{1}{2}pn_{r}^{2}+1\right)}^{e_{rr}+1}}\prod_{r<s}\frac{e_{rs}!}{{\left(pn_{r}n_{s}+1\right)}^{e_{rs}+1}} \end{array}$$ (11)

for the standard SBM or

$$\begin{array}{c} P_{\text{dc}\;\text{SBM}}\left(G|b\right)=P_{\text{standard}\;\text{SBM}}\prod_{r,n_{r}\neq 0}\frac{n_{r}^{e_{r}}\left(n_{r}-1\right)!}{(n_{r}+e_{r}-1)!} \end{array}$$ (12)

for the degree-corrected SBM. The probability *P*(*G*) is unknown but not needed for comparing partitions and unlike other formulations of the SBM this variant allows empty blocks.

As last flat variant we introduce Peixoto’s microcanonical formulation, which is the basis for two further variants of the next sections. For the standard SBM the microcanonical formulation of Peixoto [2] yields

$$\begin{array}{c} P_{\text{standard}\;\text{SBM,}\;\text{Peixoto}}\left(G,e,b\right)=P\left(G|e,b\right)P\left(e|b\right)P\left(b\right) \end{array}$$ (13)

with

$$\begin{array}{c} P(G|e,b)=\frac{\prod_{r<s}e_{rs}!\prod_{r}e_{rr}!!}{\prod_{r}n_{r}^{e_{r}}}\frac{1}{\prod_{i<j}A_{ij}!\prod_{i}A_{ii}!!}, \end{array}$$

P(e|b)=((K(K+1)/2M))−1, (14)

P(b)=∏rnr!N!(N−1K−1)−11N, (15)

where we denoted with (2*n*)!! = 2^*n*^*n*! the double factorial. The standard SBM does not depend on the degree sequence **k**, which is needed in the degree-corrected case. His formula for the degree-corrected SBM is

$$\begin{array}{c} P(G,k,e,b)=P(G|k,e,b)P(k|e,b)P(e|b)P(b) \end{array}$$ (16)

with

$$\begin{array}{c} P\left(G|k,e,b\right)=\frac{\prod_{i}k_{i}!\prod_{r<s}e_{rs}!\prod_{r}e_{rr}!!}{\prod_{i<j}A_{ij}!\prod_{i}A_{ii}!!\prod_{r}e_{r}!}, \end{array}$$

where *k*_*i*_ is the degree of node *i*. *P*(**e**|**b**) and *P*(**b**) are equal to above (14) and (15). The the probability *P*(**k**|**e**, **b**) Peixoto gives two choices

Puniform(k|e,b)=∏r((nrer))−1, (17)

$$\begin{array}{c} P_{\text{uniform}\;\text{hyperprior}}\left(k|e,b\right)=\prod_{r}\frac{\prod_{k}N_{k}^{r}!}{n_{r}!}\text{q}{\left(e_{r},n_{r}\right)}^{-1}, \end{array}$$ (18)

where $N_{k}^{r}$ denoting the number of nodes with degree *k* in group *r* and q(*m*, *n*) being the number of restricted partitions of the integer *m* into at most *n* parts.

Other approaches, proposing solution to the selection of number of groups, are based on nonparametric Bayesian model and use the Chinese restaurant process [64] or features together with the Indian buffet process to solve this task [65]. We did not include these models in our comparison. A table at the end of this section will list all included SBM variants, together with a reference to their equation, their authors and the latter used abbreviations.

#### Hierarchical SBM

Community detection methods often have a known minimum group size under which the algorithm is not capable to infer statistical significant groups. This boundary is known as a resolution limit and for the SBM variants described above, the resolution limit is $O(\sqrt{N})$. Smaller groups are typically merged together with neighboring blocks and the methods can not retrieve the true community structure. The solution to this, was to adopt the idea of hierarchical clustering methods like [66] to the concept of SBM.

**Fig 3:**
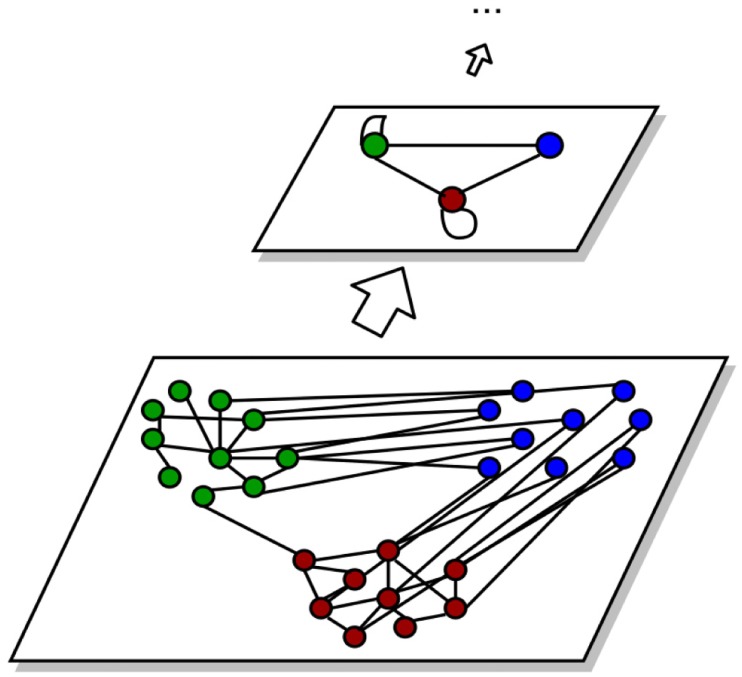
The resulting SBM of a graph can be represented as a multi-graph, which contains a node for each block and the edges between the blocks like in the underlying graph. Based on this representation of a SBM the process of inference can be iterated by applying the method again to this representation.

To allow the detection of smaller groups by lowering this boundary, Peixoto proposed hierarchical variants of the SBM [2, 37]. The result of an inference of a SBM can be represented as a multigraph with the groups as nodes and the edges given by the corresponding edges of the nodes inside each group. The idea of hierarchical SBM is that the multigraph was again generated by a SBM and with this create a hierarchy of stochastic block models like displayed in Fig 3.

To include this extension into the theory, we can adopt the probabilities for the partition *P*(**b**) and the edge counts *P*(**e**) in (13) and (16). We now use

P(e)=∏l=1L∏r<s((nrlnslersl+1))−1∏r((nrl(nrl+1)/2errl+1)) (19)

and

P(b)=∏l=1L∏rnrl!Kl−1!(Kl−1−1Kl−1)−11Kl−1, (20)

where *L* is the hierarchy depth and upper indices indicate specific level, e.g. $e^{l}=(e_{rs}^{l})$ is the (weighted) adjacency matrix at level *l*. Moreover, we set *K*^0^ = *N* and enforce *K*^*L*^ = 1.

The enhancement of the resolution limit we will test in the analysis section against the model selection of the previous section and the different choices for the classic SBM variants. With its different levels a hierarchical SBM captures a variety of scales to view the data.

#### Further extension

Beside the already presented variants of the SBM, researcher have developed further extensions to cover additional situations like overlapping nodes, valued edges or time-dependent networks. Here, we only want to give a short insight into these variants to give a broader overview of available models.

#### Multiple group assignments

If you think of your own social network, you probably belong to a number of social groups like your colleagues, your sports club and so on. Applying the SBM to the network created of your friends and their friendships would probably retrieve those groups with the exception that you would be assigned to another group, because your ties do not match with any single group. Placing yourself into all of these groups would describe the reality in a better way.

Based on this idea, the SBM variants with multiple groups break the strict assignments of nodes to single groups and allow multiple or partial assignments to groups. To distinguish between multiple binary assignments and multiple partial assignments, the first is usually referred as overlapping groups and the second as mixed membership [38, 67, 68].

#### SBM on directed graphs

Another possibility in generalizing the framework of SBM, is to drop the constraint of undirected graphs and to allow directed networks. Apart from some exceptions such as [1, 34, 39, 69], most authors assume undirected graphs for simplicity reasons and do not explicitly specify the formulas of their SBM variant for directed graphs. Therefore other have extended their concept to the directed case like in [70], where Zhu et al. generalized the degree-corrected SBM of Karrer and Newman to the directed case. As already mentioned, we included those generalization and other straight forward generalization in the same manner for the described SBM variants in our code.

#### SBM on weighted graphs

The starting point of many complex networks are real phenomena, which often contain additional information about the strength of the connections. Think for example of capacity in the power network, the number of passengers in the worldwide flight network or the strength of a friendship in a social network. But simply introducing a threshold and representing those ties as binary edges would miss important information of the observed system. Therefore, the SBM was extended again to cover weighted networks, as well [39, 40, 71].

The basic idea of extending the SBM to weighted networks is based on principle of the standard SBM. Analog to the edge distribution, the weight distribution of the edges is assumed to be the same for each pair of nodes between two groups. The research on weighted graphs is not yet as mature as the general SBM approaches and because this approaches cannot be directly compared with the other approaches, we only refer to two examples, where implementations are available.

One of the first authors, who included edge weights or covariates into the SBM are Mariadassou et al. [40]. They included a Poisson distribution for the weights in their expectation maximization algorithm. Their method was also implemented as an R package [72]. Peixoto describes a selection of different distributions for the edge weights [39], which is like his other approaches included in his Python graph-tool package.

#### SBM with metadata

Another kind of information which is usually available for networks of the real world are node attributes like the age or class of pupils. Peel used this annotated data for an supervised variant of the SBM and added an additional layer in the generative process of the SBM [69, 73]. On the contrary Zhu et al. used an extension of the SBM to use the topic mix inside documents to improve the description of links between each other [74]. Others studied to which extend the metadata is correlated with the observed topological structure [51, 75].

#### Dynamic SBM

In the fortunate but more challenging case, that multiple observations of the regarded phenomenon are available, the so called dynamic SBM can be applied. Based on the same assumption as in the other SBM variants, that the networks are generated by the generative model, the aim is to infer this structure from the networks. The degree of freedoms vary between different dynamic SBM variants. The major differences are in the handling of the interactions of the edge probabilities and communities between the observed time-steps, which can be either independent, i.e. only conditioned on the actual network, or in some way connected to other observations [76–79]. Ghasemian even proved some thresholds for community detection with SBM in dynamic networks [80].

The fundamental different assumption on the available data of variants of this section, like weight information or multiple time points, prevent a common set of networks for testing for those and the other variants. Thus, we exclude these variants from our analysis and have only included them for an complete overview of all SBM types.

### Summary

To summarize the presented variants and the different objective functions, we give an complete overview in Table 1. The included abbreviations will be used in the analysis section for an easier identification of the different variants. For the variant proposed by Côme and Latouche we will test both priors and call them ICLexJ and ICLexU according to the used Jeffrey respectively uniform distribution.

**Table 1:** SBM variants, authors and assigned abbreviations.

SBM Variant	Authors	Equation	Abbreviation
Standard SBM	Karrer and Newman	(2)	SKN
Degree-Corrected SBM	Karrer and Newman	(5)	DCKN
	Peixoto	(6)	DCP
SBM including Number of Groups	Côme and Latouche	(7)	ICLexJ & ICLexU
	Newman and Reinert	(8–11) & (12)	SNR & DCNR
	Peixoto	(13), (16) & (18)	SPC, DCPU & DCPUH
Hierarchical SBM	Peixoto	i.a. (19), (20)	HSPC, HDCPU & HDCPUH

Many inference algorithms iteratively try to improve their result by evaluating the neighborhood of the current partition. As all inference algorithms used later are of this form, we complete this overview by giving the asymptotic performance for a delta calculation of a single node movement, which is

$$\begin{array}{c} \text{SKN,}\;\text{ICLex,}\;\text{SNR,}\;\text{DCNR}\in O(K+\langle k\rangle ) \end{array}$$

and

$$\begin{array}{c} \text{DCKN,}\;\text{DCP,}\;\text{SPC,}\;\text{DCPU,}\;\text{DCPUH,}\;\text{HSPC,}\;\text{HDCPU,}\;\text{HDCPUH}\in O(\langle k\rangle ). \end{array}$$

The target functions can be divided into two classes. The execution time of the faster second group solely depends on the average degree 〈*k*〉 of the network. The runtime of the other group also increases proportionally to the number of groups K.

With this introduction of many SBM variants from the standard SBM over hierarchical SBM to dynamic SBM, we have shown the variety of the stochastic block model and created a solid basis for the later comparison of the different approaches and the corresponding inference methods.

## Inference methods

Knowing the SBM variants and the corresponding likelihood functions, we need methods to approximate an optimal community structure of the selected model and in most cases the authors present a combination of a new SBM variant with a corresponding inference algorithm. However, many inference algorithms do not depend on the specific variant. Therefore, we clearly separate these two parts of the community detection problem.

Using the term of Young et al. [8], this separation is possible for all canonical algorithms, which split the task into defining a total ordering of the partitions and a maximizer, which can find (local) optima. Each objective function from Table 1 (together with their model selection) induces for a given network *G* = (*V*, *E*) a total order on the set of partitions of *V*. In other words, for any two partitions we know, which one describes the network better under the respective model, by evaluating the function. The task of the inference algorithm is simply to retrieve a good approximation of the greatest element. We restrict our analysis to these canonical formulations to allow the differentiation of effects brought by SBM variant and inference method.

We start with the simple Markov chain Monte Carlo method and continue with an agglomerative algorithm from Peixoto, local heuristics, and end with an overview of further methods like the usage of semidefinite programming.

### Markov chain Monte Carlo methods

The Markov chain Monte Carlo (MCMC) methods are a class of algorithms used to sample from a probability distribution. This can be used to sample from the space of all node partitions, where each partition is hit with its respective likelihood. These algorithms have the advantage of an easy implementation and the result usually converges in the limit to the desired distribution. Because of these advantages, various authors used this method with only minor variations [36, 63, 81].

The easiest algorithm of this class is the Metropolis algorithm. The algorithm requires only a function *F*, which is proportional to the desired likelihood, and a number of steps *k*. As described in Algorithm 1, the Metropolis algorithm suggests random moves, accepts any move which improves the function *F*, and accepts all other moves with the probability exp(−*β*Δ*F*). The so called inverse temperature *β* controls the likelihood of negative moves and can be used for simulated annealing. Simulated annealing increases the value of *β* step by step to increase the chance to stay in a local optimum at the end, but leave local optima in the beginning. The latter is a common advantage of all MCMC algorithms: the ability to perform negative moves and leave local optima. A disadvantage of this algorithm is that the correct selection of the number of steps is difficult, which is usually only bounded by the available running time.

**Algorithm 1** Metropolis algorithm

1: **for** *j* = 0 **to** *k* **do**

2:  Get a random move (*i*: *r* → *s*) of node *i* from block *r* to block *s*

3:  Calculate the improvement of the target function Δ*F*(*i*: *r* → *s*)

4:  Accept the move (*i*: *r* → *s*) with probability *p*_*A*_ = min (1, exp (*β*Δ*F*))

5: **end for**

Another MCMC algorithm is the Metropolis-Hastings algorithm, which improves the proposal of random moves with the introduction of a proposal density function *Q* and changes in the acceptance probability. The algorithm has to keep the properties of ergodicity, i.e. all states are possible, and detailed balance, i.e. all moves are reversible after a sufficient long time, to eventually reach the respective distribution. We have implemented the node proposals of Peixoto [36], which selects a random node and a new block based on the actual edge matrix and the neighborhood of the node. As first adoption, the likelihood of a move of a node from block *r* to block *s* is instead of 1/*K*

$$\begin{array}{c} p\left(r\to s|t\right)=\frac{e_{ts}+\varepsilon }{e_{t}+\varepsilon K}, \end{array}$$ (21)

where *t* is the block of a random neighbor. The parameter *ε* > 0 ensures that all moves are possible and the fully random moves are recovered by *ε* → ∞. The Metropolis-Hastings algorithms has a typical form of acceptance probability, which is for the move of the node *i* from block *r* to block *s*

$$\begin{array}{c} p_{A}=\min(1,\text{exp}\left(\beta \Delta F\right)\frac{\sum_{t}p_{t}^{i}p\left(s\to r|t\right)}{\sum_{t}p_{t}^{i}p\left(r\to s|t\right)}) \end{array}$$

with $p_{t}^{i}$ being the fraction of neighbors of *i* belonging to block *t*. The probabilities *p*(*r* → *s*|*t*) and *p*(*s* → *r*|*t*) are calculated with different values of *e*_*rt*_ and *e*_*t*_. The former with the values of the current state and the latter with the new values after the proposed *r* → *s* move.

With this we have described the basic variants of the MCMC methods, which are easy to implement and fast in the execution of a single step. But these approaches have the problem of detecting the convergence of the MCMC or, in other words, the right choice of performed number of steps.

Unless we explicitly state otherwise, we used the MCMC methods with *β* = 1 and applied no form of cooling like simulated or abrupt heating. With this choice these algorithms rather sample from the space of partitions with the probability distribution given by the objective function and leaves even the global optimum with a certain probability. We tested two simple variants of simulated variant, where the temperature *β*_*k*_ at the *k*^th^ step is given by $\frac{1+k}{T_{0}}$ respectively $\frac{log(1+k)}{T_{0}}$. We determined the initial temperature *T*_0_ by evaluating the objective function on 20 random partitions and set *T*_0_ to 1.5 times the maximal observed deviation. Since these two variants led to a deterioration of the results, we only included the variants with *β* = 1 into the analysis. Yet, elaborated variants of simulated annealing may improve the quality, but such exploration is beyond the scope of this publication.

### Agglomerative algorithm

Another approach in the style of a “bottom-up” hierarchical clustering is the agglomerative algorithm of Peixoto [36]. Like in hierarchical clustering [11], we start with each node in its own cluster and subsequently merge clusters. Contrary to hierarchical clustering, we calculate only a certain amount of *n*_mergers_ merges for each block and perform a certain ratio of the best found mergers, instead of calculating all possibilities and performing only a single merge. This reduces the time complexity of the algorithms by a huge factor and turns the algorithm into an heuristic. To allow some free movement of the nodes in between the block mergers, a certain amount of Metropolis-Hastings steps are performed. In total we have the steps of algorithm 2.

The free parameters, which tune the algorithm between being a greedy heuristic and a slower and more accurate method, are the ratio *σ* of mergers, the number of tested mergers per block *n*_mergers_, and the number of steps *τ* in the Metropolis-Hastings algorithm. In addition, the inverse temperature *β* of the Metropolis-Hastings algorithm can be used for simulated annealing or abrupt heat up of *β* → ∞ like proposed by Peixoto. We use a similar configuration to Peixoto with *σ* = 2, *n*_merges_ = 10, *τ* = 200 and an abrupt heating like described in algorithm 2.

The application of this algorithm requires on the one hand the ability to calculate the delta of the likelihood function for the merger of two blocks instead of a simple node move. On the other hand, for an efficient sampling of new blocks with the probability *p*(*r* → *s*|*t*) like described above the saving of edges adjacent to each block is needed, which adds an extra space requirement of *O*(*E*). The main advantage of this algorithm is that the running time does not depend on **K** and unlike the MCMC methods the algorithms stops when it has reached a certain result.

**Algorithm 2** Heuristic agglomerative algorithm from Peixoto [36]

1: Initialize by putting each node in its own cluster

2: **while** *K*_actual_ > *K*_aim_ **do**

3:  **for** Block *r* = 1 **to** *K*_actual_ **do**

4:   **for** *i* = 1 **to** *n*_mergers_ **do**

5:    Select a new random block *s*, where the block *s* is selected with probability *p*(*r* → *s*|*t*) (based on a random neighboring block *t*, see Eq 21)

6:    Calculate the change of the objective function Δ*F*(*r* → *s*), if block *r* and *s* would be merged

7:   **end for**

8:  **end for**

9:  Perform the $\frac{K_{\text{actual}}}{\sigma }$ block merges with the highest Δ*F*(*r* → *s*) values

10:  Apply the Metropolis-Hastings algorithm *τ*/2 steps with *β* = 1

11:  Apply the Metropolis-Hastings algorithm *τ*/2 steps with *β* = *β*_high_ = 100 000

12: **end while**

### Local heuristics

Another type of algorithm uses local optimal moves and therefore always stops in a local extreme. A member of this class is the algorithm proposed by Karrer and Newman together with their degree-corrected SBM variant [32] and used by Zhu et al., too [74]. It is similar to the Kernighan-Lin algorithm for the minimum-cut graph partitioning [82].

The algorithm identifies the overall best move of a node to a new block with the only restriction that each node can only be moved once per iteration. This heuristic results in some negative moves before all nodes are moved. Before a new sweep of all nodes is started, the visited states are searched and the state with the best value is set as starting point. The algorithm halts if one iteration finds no further improvement. A complete description is available in algorithm 3.

**Algorithm 3** Kernighan-Lin algorithm [82]

1: Start with a random partition of all nodes

2: **repeat**

3:  Calculate the values Δ*F* for all node moves and set *V*′ = *V* and $g=\vec{0}$

4:  **for** *j* = 1 **to** N **do**

5:   Retrieve the move *i*: *r* → *s* with the maximal Δ*F*

6:   Set *g*_*j*_ = Δ*F* and perform this move

7:   Set *V*′ = *V*′\{*i*} and for all nodes in *V*′ update the deltas

8:  **end for**

9:  Find *k* which maximizes $g^{k}=\sum_{\alpha =1}^{k}g_{\alpha }$

10:  **if** *g*^*k*^ > 0 **then**

11:   Go back to the corresponding state

12:  **end if**

13: **until** *g*^*k*^ ≤ 0

One iteration has a running time of *O*(*N*^2^*K*) and the algorithm usually stops after a few iterations. The running time is mainly influenced by the calculation of the best possible move. As part of our evaluation, we used the full version only for the smaller networks and therefore used two variants of this approach, which have an acceptable runtime for all used graphs.

As a first modification we tried to break up the strong need of updating all deltas. Now we simply iterate through all nodes and determine the best move for each node or no move if no improvement is possible. In a second step we perform all moves and the advantage of this procedure is that the first part can be done in parallel. In the other variant, we iterate again through all nodes and now always perform the best available move for each node. These two variants should be a lot faster then the original algorithm, but these adaptations may result in inferior local optima.

All the presented local heuristics are deterministic in their execution and should be applied multiple times from different starting partition to retrieve a better result.

### Further algorithms

This list of algorithm is far from being complete and most algorithms which aim to minimize some sort of target function by switching nodes between groups, like the ones for modularity optimization, can be applied. We concentrated on those algorithms proposed in the context of SBM or its variants, which can be applied independently of the concrete variant. In this section we want to discuss some other approaches that are more strongly linked to formulation of the variant and that have been proposed in the context of SBM variants.

#### Belief propagation and spectral algorithms

Some authors try to prove thresholds to which the community detection problem can be solved. Two approaches often used in these publications are belief propagation and spectral algorithms.

Spectral algorithms are based on the feature that eigenvalues and their corresponding eigenvectors of specific matrices are connected to the partition of nodes. The spectral algorithms consist of creating the specific matrix from the network, calculating the *K* leading real eigenvalues and solving a clustering task in ℜ*^K^* with methods like k-mean. Therefore, the community detection problem can be transferred to linear algebra and usual clustering, where fast and efficient methods are available. The difference between the spectral approaches lies in the usage of different matrices. Starting from the adjacency matrix, several authors proposed variants with (normalized) Laplacian, modularity matrix, random walk matrix or nonbacktracking matrix [83, 84].

Another approach used to achieve thresholds, are variants of the belief propagation or message passing algorithm [17, 21, 26, 28, 80, 85]. In those algorithms nodes send so-called messages to their neighbors, consisting of estimates of their marginal distribution. Each node uses the received messages to update its own estimation. The process of sending and updating estimations is repeated until a fixed point is reached. If the parameters of the SBM are not known, the fixed-point iteration is alternated with updates of these parameters. Zhang et al. [26] proposed to use the result of a spectral algorithm as starting point for the belief propagation to accelerate convergence and improve results.

#### Expectation-maximization algorithm

Another approach often used together with belief propagation is the expectation-maximization (EM) algorithm. With its alternating sequence of updating two sets of parameters, the EM algorithms retrieve a local optimum [17, 86].

Beside the usages together with belief propagation, the EM algorithm was used in early works by Snijders and Nowicki [53] and is often applied to the mixed membership variant of the SBM, where the node assignment to groups is not binary, but continuous [68].

#### Semidefinite programming

A different formulation of the inference problem is the perspective of semidefinite programming (SDP). In SDP the inference task is stated as an objective function in the form of a inner product and constraints like semidefiniteness constraints on matrix variables. For example, the formulation in Yan et al. [87] is

$$\begin{array}{cc} \max & \;\text{trace}(AX)-\lambda \text{trace}(\lambda X)\\ \text{s}.\text{t}. & \;X⪰0,\;X\geq 0,\;X1=1, \end{array}$$

where λ is a tuning parameter, which is determined by its own heuristic, and the constraints on clustering matrix *X* are positive semidefiniteness, non-negativity, and the summation of each row to 1. As far as we see, this approach was applied from different authors for the standard SBM, but not for any of the variants. Some of the work is even limited on the detection of two clusters with the aim to lower realized detection thresholds of exact recovery in the planted bisection model [18, 87, 88].

Because the methods are only available for standard SBM and include their own model selection technique for the correct number of blocks, we have not included the algorithm of Yan et al. in the comparison of standard SBM versions and in the corresponding analysis of model selection techniques.

### Summary

Like previously for the SBM variants, we want to restate the presented inference algorithms, assign abbreviations for the analysis, and take a first look at the theoretical performance.

Metropolis algorithm (MA) and Metropolis-Hastings algorithm (MHA) have both no termination condition and so their execution time is solely a product of the given number of steps and the cost for calculating the delta of a proposed node move. Unfortunately, this crucial parameter is often not stated in publications. As an orientation we only have the numbers published by Newman and Reinert, which was between 10.000 steps for small and 100.000 steps for larger graphs [63].

Next, we have described Peixoto’s agglomerative heuristic (PAH), which has according to Peixoto a time complexity of *O*(*N* ln^2^ *N*) [36]. The outstanding property of this algorithm is its independence on the number of inferred blocks K.

As a last algorithm in our analysis, we have described the Kernighan-Lin algorithm and two of its variants. The original algorithm will be named KL. The version applying the search for each node first and afterwards performing an update of all node position will be referred as KL-EM, because of its analogy to the principle used in EM algorithm. The second variant with the direct update for each node is denoted KL-G because of its greedy nature. The runtime complexity for all these variants depends on the number of iterations needed until convergence. But the required number of calculated deltas for an iteration can be stated with *N*^2^ *K* for KL and *NK* for KL-EM and KL-G.

With this non exhaustive overview of inference methods, we have prepared a set of variants as well as methods for our analysis.

## Comparison of SBM variants and inference methods

For a systematic test of the presented variants and inference methods we need a suitable set of networks with a known community structure of different strength. The Girvan-Newman test and the LFR test define such sets consisting of random networks generated based on a known node partition and predefined mixing parameters [10, 11]. These tests allow us on the one hand, to compare the performance of SBM variants and inference methods and on the other hand, the comparison to other clustering techniques. To demonstrate applicability and suitability in real cases, we conclude this section with a selection of real networks.

One consequence of the approximate no free lunch theorem [14] is that all methods perform equal, if we average over all cases. On subsets, methods can still be better than others, but this is correspondingly exactly complementary on the respective complement of the subset. We want to compare the methods on a natural subset that is given by those artificial networks of the detectable region of parameters. As another remark, we can not answer to what extent the task of the inference algorithms to maximize the objective functions for these networks is affected by the no free lunch theorem for search and optimization [89].

### Girvan-Newman test

Girvan and Newman used a set of computer generated networks to compare their proposed clustering algorithm [11]. This test was later adapted by other authors and named according to the authors either Girvan-Newman test or GN test [22, 90].

The test consists of networks generated with the planted l-partition model. This model is characterized by the groups, the probability *P*_in_ for an edge inside a group, and *P*_out_ the probability for an edge between two groups. The planted partition model can be viewed as a reduced version of a standard SBM, where we restrict the edge probability matrix *ω* to have only two different values *P*_in_ and *P*_out_, the probability for an edge inside a block respectively an edge between blocks. In the GN test, the number of groups is fixed to four with each consisting of 32 nodes. The proposed algorithm of Girvan and Newman aimed only for assortative communities, so they only regarded *P*_out_ < *P*_in_. The probabilities were chosen in a way that the average degree is 16 for all networks. With this we have one free parameter left to vary.

We have extended the test set of the GN test to the range of probabilities with *P*_out_ ≥ *P*_in_. Our main interest is to determine to which extent assortative and disassortative community structures are detectable.

Because Girvan and Newman used deterministic algorithms, they generated 100 network instances for each parameter. In our setting we use algorithms which are at least non deterministic in their dependency on the random starting position. We apply every combination of algorithm and SBM variant 10 times to 10 network instances for every parameter, which results in the same amount of executions per combination. First, we analyze the SBM variants under the assumption of knowing the true number of groups and then compare the inference algorithms, before comparing the model selection.

The comparison of inferred partition and planted partition needs a function, which evaluates their similarity. For this task numerous solutions exist like the old-fashioned fraction of vertices classified correctly [11, 22] or normalized mutual information [13, 22]. We decided to take the adjusted mutual information (AMI) [91], which has the advantage to include a correction for chance and is normalized (in a stochastic sense) to [0, 1]. To be exact, we use the AMI_max_ version of [91] and will always simply call it AMI. Gates and Ahn discussed the influence of the normalization method (like max) and the chosen random model for the correction for chance [92]. Following their results, we could use a random model with fixed number of clusters in those cases, which includes only partitions with a fixed number of groups. But we have decided to always use the [91] variant to facilitate the comparison of our results between the different cases considered.

**Fig 4:**
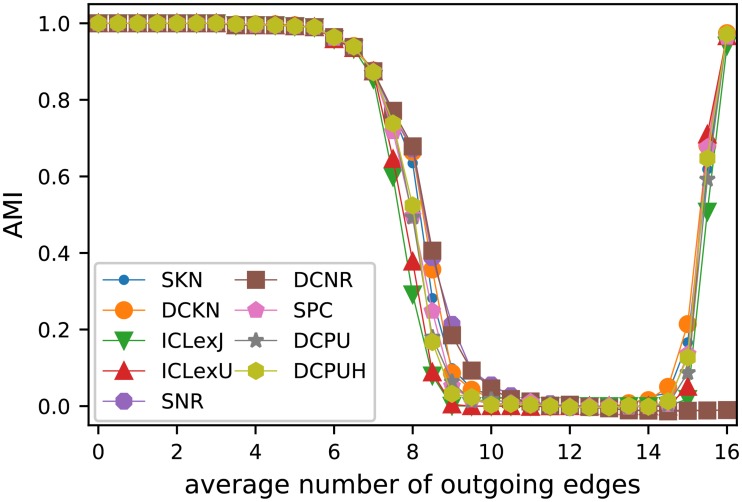
Results of Girvan-Newman test applied with a known number of groups. Each marker represents the average of 10 network instances. For each network each of inference algorithm (see Fig 5) were executed 10 times from random partitions with 4 blocks for each of the shown SBM variants. The AMI of the partition with the best objective value of all inference algorithms and all executions is taken into account. Since the results of the DCP algorithm are the same as the SKN for the case without model selection, the diagram only includes the results of SKN.

For the comparison of the SBM variants, we decided to select for each network and variant only the partition of nodes with the highest objective value (likelihood) of the respective model. Because we only use the likelihood for the selection, such an approach is also applicable in any real situation. The results in Fig 4 show a high consistency between all SBM variants. Until a value of *k*_out_ = 6 outgoing edges, the true structure is retrieved in nearly all cases and starts to diminish afterwards reaching its minimum at *k*_out_ = 10. From *k*_out_ = 15 most variants are able to detect the now disassortative structure again and only the two variants proposed by Newman and Reinert fail in these cases.

**Fig 5:**
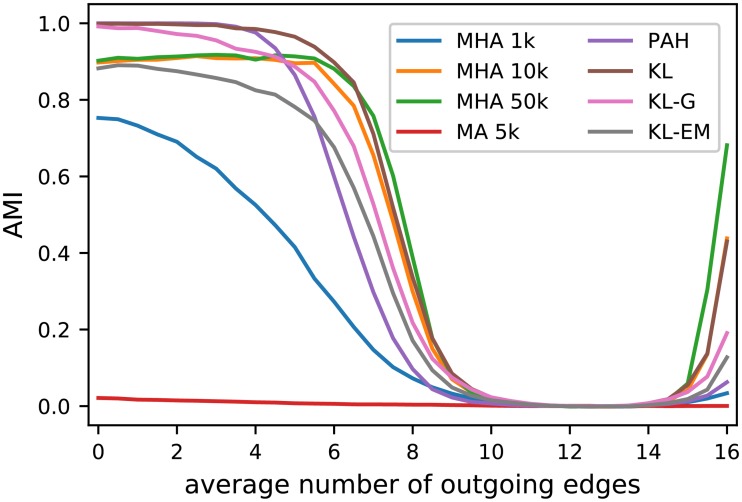
Overview of the performance of the applied inference algorithms. The results shown are based on the same 10 executions for each of the 10 networks for each SBM variant of Fig 4, which are the basis for Fig 4. The value shown are the mean of all executions, all networks with the same *k*_out_ and all SBM variants (of Fig 4). Fig 6 includes the results for selected inference algorithms for individual SBM variants.

Knowing that all SBM variants are applicable in this scenario, we decided to compare the inference algorithms over all SBM variants. Fig 5 shows the obtained results. Each point in the diagram corresponds to the mean of 900 executions. The simple Metropolis Algorithm (MA) delivers the worst inference results by far. Its pure random proposal of node moves led to only few accepted moves. The move proposals in the Metropolis-Hastings Algorithms (MHA) clearly outperforms MA, which can even be seen in the case of only 1 000 moves (MHA 1k). The results can be improved further by increasing the number of steps to 10 000 or 50 000, which increases the average performance in complex scenarios a lot. But, still multiple executions with fewer steps can outperform a single execution with a high number of steps.

Peixoto’s agglomerative heuristic (PAH) performs consistently good for simple cases, but starts to fail earlier regarding both the average and the best retrieved results. The Kernighan-Lin algorithm (KL) and both variants (KL-G, KL-EM) retrieve good results. The expectation maximization alike KL-EM variant delivers the worst results of all tested variants and only the full KL algorithm is able to retrieve steady, near to perfect results until *k*_out_ = 6.

**Fig 6:**
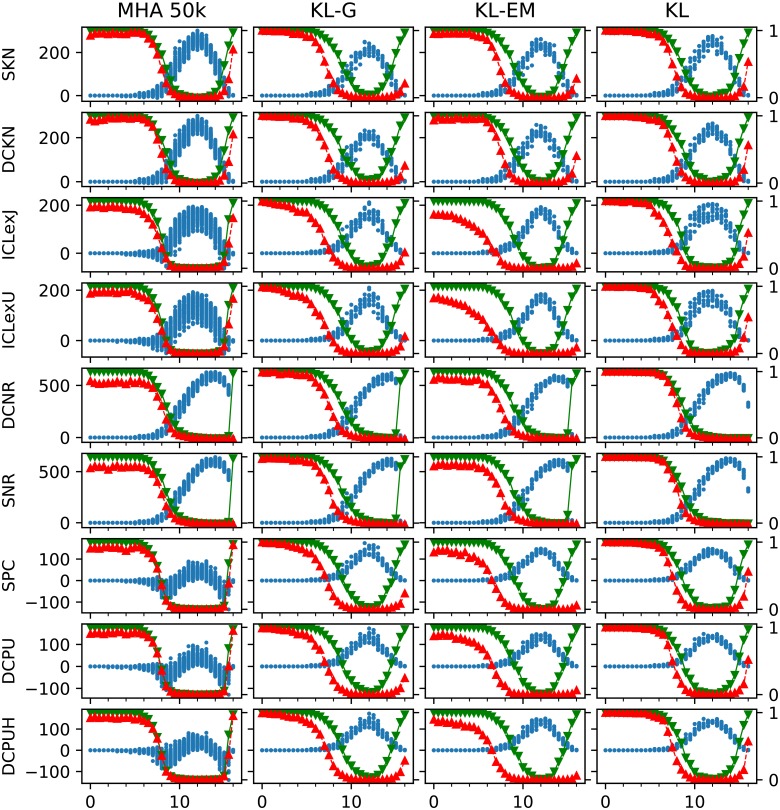
Results from starting the inference algorithms from the true partition 10 times for 10 network instances for each *k*_out_ = 0, 0.5, …, 16. The resulting difference to the original value (designated with a blue dot ●), the average AMI of all runs of all network with the same parameter (represented with green triangles upside down ▼) and for comparison the average in the same manor reached from a random starting partition with the known number of groups (designated with red triangles ▲) are shown. The AMI values (▼,▲) of each row are visualized according to the right axis of each row. The difference of the objective functions we calculate with *P*_observed_ − *P*_true_ and is measured according to the left axis in each row. If the regarded model describes a likelihood, positive values represent more likely partitions then the planted partition. Each small diagram contains the values of the model of its row and the inference algorithm of its column. To reduce the total number of diagrams shown only the results of the Metropolis-Hastings algorithm with 50 000 steps is shown. Because PAH is not designed to start from a given partition, it is not included, too.

Before taking a look at selecting the numbers of group, we try to isolate the contribution of the model and the algorithm. Therefore, we tested the optimal starting partition and executed the algorithms with each model 10 times on each of the 10 network instances for *k*_out_ = 0, 0.5, …, 16. Fig 6 shows the results of these executions. As expected the ideal starting partition improves the results in all combination. The results of the MHA 50k confirms, that the planted partition is a stable optimum up to *k*_out_ = 6. Beyond this interval the models differ in their behavior. The models ICLexJ, ICLexU, SPC, DCPU, and DCPUH first start to equilibrate at likelihoods slightly below the one of the planted partition, before taking higher values like the others. The executions of KL-G and KL-EM show, that new optima exist for 15 > *k*_out_ > 6. In this region the respective partitions of these optima describe the planted partition first in decreasing quality and then from *k*_out_ = 12 the quality of these partitions increases again with the same slope. The only exceptions of this behavior are the models SNR and DCNR, which for MHA 50k, KL-G and KL-EM do not show a steady increase for *k*_out_ > 12, but a discontinuously increase for *k*_out_ > 15. Taking the results of KL for SNR and DCNR into account, we see, that even for *k*_out_ > 15 those model have another higher maximum beside the values. This explains the bad performance of these models for *k*_out_ > 15 in Fig 4, where only the AMI of those partitions with the maximal observed value are included.

**Fig 7:**
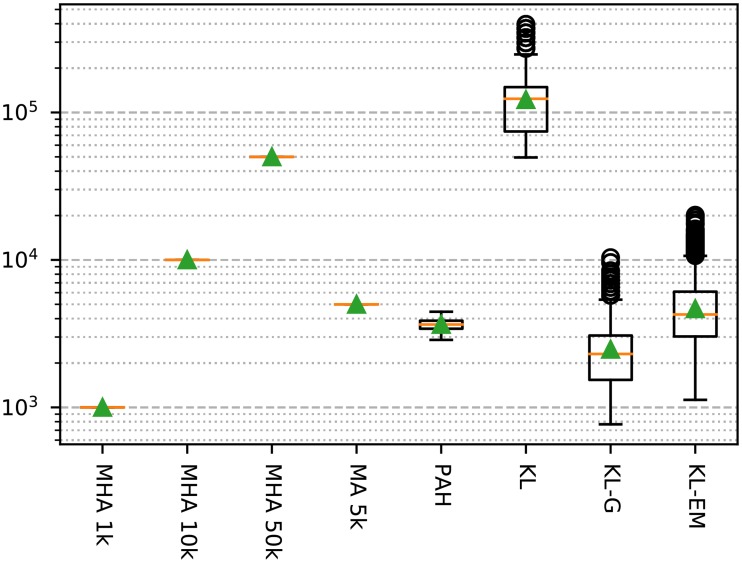
The recorded number of calculated deltas of the studied inference algorithm during all performed runs for Fig 4. The green triangle marks the mean and the orange line represents the median of the values.

The decision which inference algorithms to apply for the model selection requires information about their efficiency. We have selected a specific measure, which is shown in Fig 7. During the inference we recorded the number of calculated deltas, number of node moves, and the time required for each execution of the inference algorithms. The advantage of the first two measurements is their independence from the used implementation. The Kernighan-Lin algorithm requires with an average of around 125 000 delta calculations the maximum of steps. All methods based on the metropolis algorithm require exactly the amount given by the number of steps. The remaining algorithms need comparable amounts of calculations, but some of them have other overheads like the block merging for Peixoto’s algorithm (PAH), or the stop criterion in both KL-variants. Taking the results in Figs 5 and 7 into account, we decided to use the Metropolis-Hastings Algorithm with 50 000 steps for the analysis of the model selection. This has the additional advantage of the run time independence with regard to the number of blocks.

In the above executions we have used the knowledge of the correct number of blocks. Now we want to compare the model selection techniques and the SBM variants, which also includes this selection step. We have again executed all objective functions with the chosen inference algorithm for the range of number of blocks from 1 to 10 and selected the optimal structure. Like above, we used 10 network instances for each *k*_out_ and applied the inference algorithm 10 times for each block size and network.

**Fig 8:**
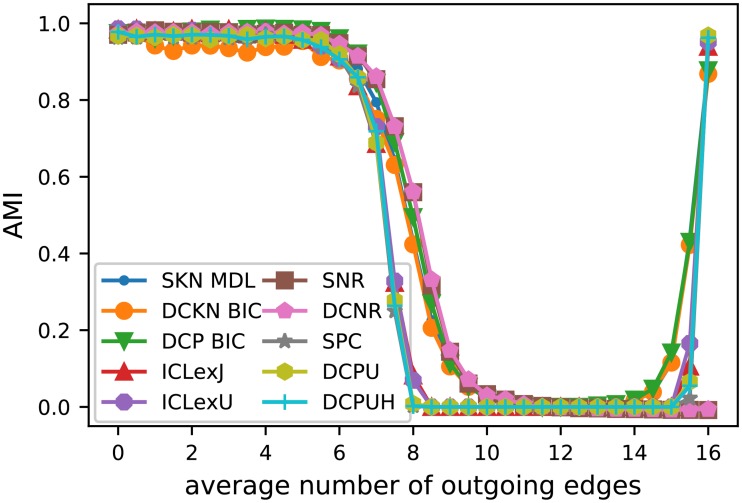
Results of Girvan-Newman test with model selection. Like before, we used 10 network instances for each *k*_out_. For each network and each SBM variant we executed the MHA 50*k* 10 times for each *K* = 1, …, 10. Then we considered one execution for each network for all number of group *K* ∈ {1, …, 10} as one unit and executed the model selection based on these results. Therefore, a data point is the average of 100 AMI values resulting from the 10 network instances and the 10 selected partitions. For the classic models only the best model selection according to Table 2 is shown.

Fig 8 shows the average AMI of the different SBM variants after performing the model selection. For the classic SBM, Fig 8 contains only the model selection with the best values for *k*_out_ ≤ 8, while Table 2 includes the rationale for our selection. The results with model selection are a little bit more scattered than the results with the known group size, but still have the same overall behavior.

**Table 2:** Normalized AUMIC of the different SBM variants of the GN test for 0 ≤ *k*_out_ ≤ 8 based on 10 executions of the MHA 50 000 from 10 random partitions for each group size *K* = 1, …, 10 and each *k*_out_ = 0, 0.5, …, 8.

Variant	True Group Size	With Model Selection	
SKN	**0.91**	MDL	0.89
		AIC	0.56
		BIC	0.84
DCKN	**0.91**	MDL	0.75
		AIC	0.56
		BIC	0.87
DCP	**0.91**	MDL	0.88
		AIC	0.58
		BIC	**0.91**
ICLexJ	0.82		0.81
ICLexU	0.83		0.81
SNR	0.81		0.84
DCNR	0.80		0.84
SPC	0.86		0.81
DCPU	0.87		0.82
DCPUH	0.87		0.81
HSPC	–		0.57
HDCPU	–		0.59
HDCPUH	–		0.56

**Fig 9:**
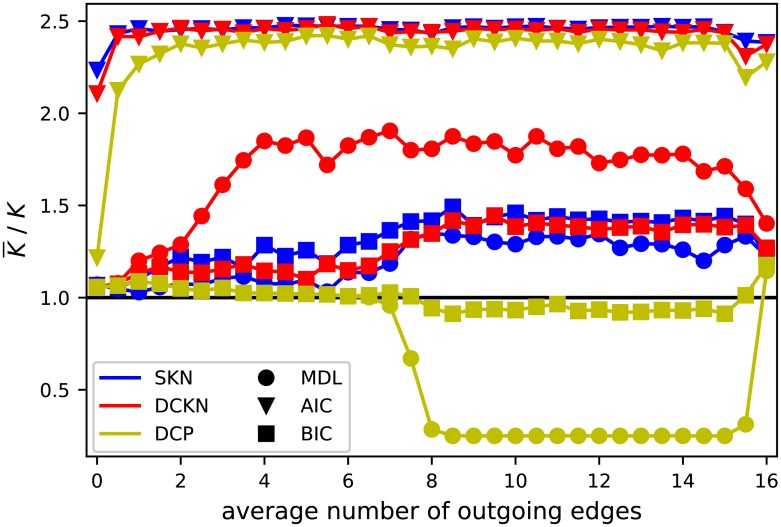
Ratio of average selected number of blocks $\bar{K}$ to true number of blocks *K* of the classic SBM variants with the presented model selections. As in Fig 10, the values shown are based on the same setting as Fig 8.

**Fig 10:**
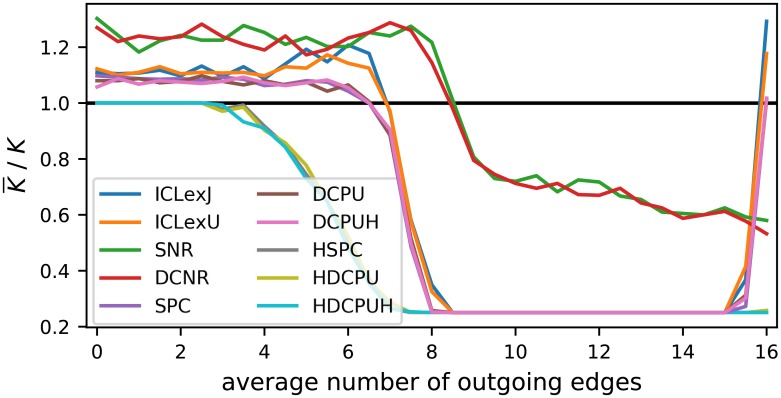
Ratio of average selected number of blocks $\bar{K}$ to true number of blocks *K* of those SBM variants which include model selection step. The values shown are based on the same setting as Fig 8.

Beside the resulting AMI of the inferred partitions, we take a look at the number of groups selected. Therefore, Figs 9 and 10 contain a visualization of the selected number of groups as ratio of the average selected value and the true value. This reduction gives us a good overview and is a valid method as long as all values tend to be relatively close to the average, which is true in this case. In general, the upper bound is given by the maximal tested number of blocks, which was 10 and, respectively, as ratio 2.5. The lower bound represents a partition of all nodes in a single block. The selection of a single block is a rejection of any found structure under a point of statistical significance in favor of a pure random Erdős-Rényi graph.

The classical SBM variants in Fig 9 show a broad range of results. The AIC overestimates the number of groups in any case and like reasoned above, the requirements of the AIC are not met. Consequently, it should not be used in the case of SBM. The BIC has for the variants of Karrer and Newman the slight tendency of overfitting, i.e. selecting too many groups. The combination of Peixoto’s degree-corrected variant and BIC is capable to retrieve the true number of groups in nearly all cases. However, since it also recognizes structure in unidentifiable cases such as k = 12, this is not very reliable. The only combination which clearly rejects a noisy structure for the simplest model is the combination of DCP and MDL.

The newer SBM variants retrieve good results and all but the variants SNR and DCNR reject the structure in the critical region. The hierarchical variants infer the true structure with the true number of groups for a small number of edges between the groups, but are faster in rejecting the structure.

To conclude with an short overview of the results and to allow an easier comparison, we propose a measure inspired by the area under the receiver operator curve (AUROC) used in machine learning by calculating the area under the AMI curve (AUMIC). In contrast to the AUROC, the AUMIC does not have a probabilistic interpretation. Yet, the AUMIC as kind of averaged AMI result can be useful for comparison as long as we select a region of the integral in a way, that the AMI curves are monotonic functions. Based on our experimental results (see Fig 4), this requirement is satisfied for 0 ≤ *k*_out_ ≤ 8 in the case without model selection. To minimize the effect of random fluctuations, we created the curve like in Fig 8 as average of the 10 executions with the MHA 50 000 for the 10 networks for each *k*_out_ = 0, 0.5…, 8. We calculated the AUMIC for one of the SBM variants with a numeric integral of the selected region by applying the trapezoidal rule to the respective AMI values and afterwards normalized the value by maximal possible integral (e.g. 8 in the case of the GN test).

To differentiate the influence of model selection, we calculated for each variant two AUMICs, one with the known number of groups and one after applying the model selection. For easier comparison with other cases we normalized the results by maximal possible area, which allows us to compare the results between two benchmarks. For a fair comparison all values in Table 2 are based on the 10 executions of the MHA 50 000 algorithms used for the model selection. Only the outcome of the hierarchical variants (HSPC, HDCPU, HDCPUH) were generated using an adopted version of Peixoto’s agglomerative heuristic, which needed a comparable amount of delta calculations in our experiment. During interpretation of the AUMIC we should keep in mind the desirable feature of some variants to refuse weak structures, which will worsen their corresponding AUMIC.

According to the resulting AUMIC presented in Table 2, the SBM variants without model selection have the highest AUMIC in the case of the known group sizes. From the variants including model selection Peixoto’s flat variants (SPC, DCPU, DCPUH) performed best. Taking the model selection step into account, the results of the old variants (SKN, DCKN, DCP) depend highly on the choice of model selection, which agrees with our findings based on Fig 9. The hierarchical variants deliver the poorest results, which is mainly caused by the earlier rejection of the structure see Fig 10, and may influenced by the assigned small execution time. The other SBM variants with inclusion of the model selection roughly have the same quality on this aggregation level and for this benchmark.

### LFR benchmark

The GN test is a simple and fast way to get a basic comparison and can be used to concentrate further tests on most promising approaches. But the contained networks do not represents most real cases with their equal sized communities, narrow degree distribution and relatively small network size. Therefore, Lancichinetti, Fortunato and Radicchi developed a benchmark for testing community detection algorithms, that is known as LFR benchmark [10, 13, 93, 94].

In contrast to the GN test, the degree distribution and the community sizes in the LFR benchmark are both drawn from a power law distribution. For both distributions, minimal and maximal values are selected and the average degree is chosen. Like in the GN test, the fraction *μ*_*t*_ of edges inside groups and total edges are varied to test community detection algorithms. Because of the rewiring step used to ensure the mixing between internal and external edges, the edges are not conditionally independent. Thus, the resulting networks are only similar but not identical to ones generated by a degree-corrected SBM, where the node degrees and community sizes follow different power laws, and the fraction of external links and the connection matrix are fixed.

We have used the setup given in [10] with 1000 nodes, an average degree of 20, a maximum degree of 50, −2 as exponent of the degree distribution, and −1 as exponent for the community size distribution. Community sizes are chosen between 20 and 100, resulting in 14 to 23 clusters in our networks. As in the GN test, we generated 10 networks and varied the mixture parameter *μ*_*t*_ from 0 to 0.6. We generated the networks using the code supplied in the original publication [93]. Each combination of algorithm and SBM variant is applied 10 times with different random starting partitions for each network.

**Fig 11:**
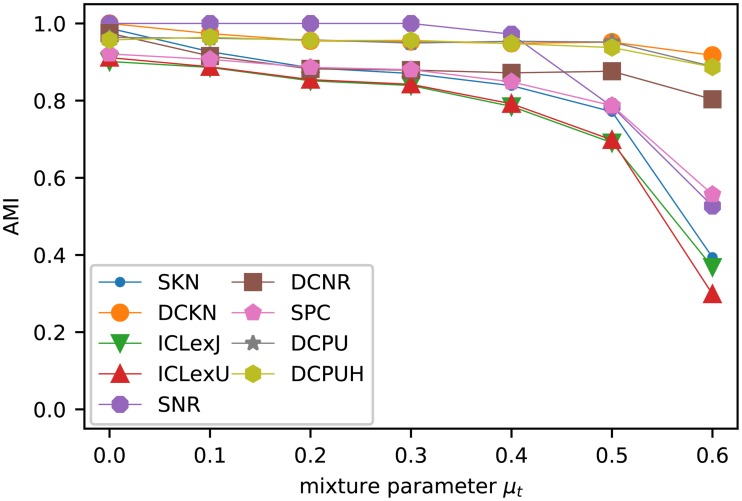
Results of LFR benchmark applied with the known number of groups. Each marker represents the average of 10 network instances. For each network each of inference algorithm (see Fig 12) were executed 10 times from random partition with the same number of blocks as the planted partition for each of the shown SBM variants. The AMI of the partition with the best objective value of all inference algorithms and all executions is used as result for the combination of network and model. Since the results of the DCP algorithm are the same as the SKN for the case without model selection, the diagram only includes the results of SKN.

First, we discuss the optimal results of different variants retrieved with the known group size and take a look at the model selection step of the group size later. Fig 11 shows our results and unlike the GN test, the different variants now inherit different qualitative characteristics.

Because the structure of the LFR benchmark is closer to the generative model of the degree-corrected variants, we had assumed a better performance of the degree-corrected variants in comparison to their non-degree-corrected counterparts. Furthermore, the number of groups may lie above the known theoretical threshold, that should be an additional advantage of the hierarchical variants. Surprisingly, the standard SBM variant SNR infers the true partition with near to 100% for *μ*_*t*_ ≤ 0.4 and outperforms all other variants in this region, if the group size is given. Only beyond this value its results decline below those of the degree-corrected variant.

In the more difficult region with *μ*_*t*_ > 0.4 the degree-corrected variants DCKN, DCNR, DCPU, and DCPUH retrieve good results and the oldest degree-corrected variant (DCKN) has the best results. The variants ICLexJ and ICLexU from Côme and Latouche exhibit on the other side the worst results. All other standard variants perform in between.

**Fig 12:**
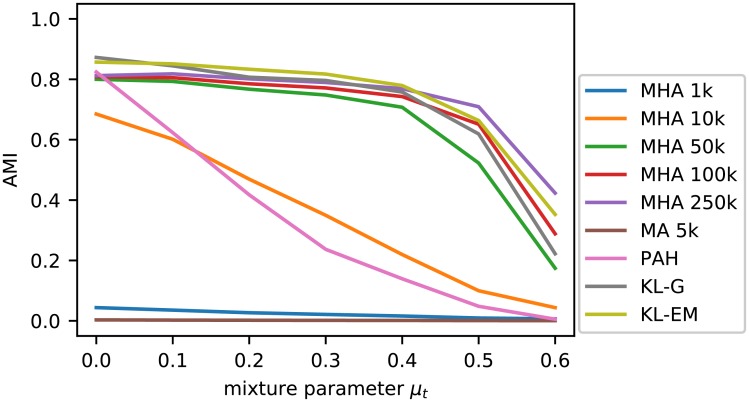
Overview of the performance of the applied inference algorithms. The results shown are based on the same executions as Fig 11, i.e. 10 executions for each of the 10 network instances for each SBM variant. The value shown are the mean of all executions with the same *μ*_*t*_ and all SBM variants (of Fig 11). Fig 14 includes the results of selected inference algorithms for individual SBM variants.

Now, we want to take a look at the performance of the inference methods. In Fig 12, we see again a similar shape of most inference algorithms. KL-G and KL-EM are the best methods and KL-EM slightly outperforms KL-G in the most difficult case with *μ*_*t*_ = 0.6. Only a little bit inferior are the results of the Metropolis-Hasting algorithm with 50k steps, which is now a lot better than those with 10k steps. In this larger test case PAH retrieves relative poor results, which can be improved by increasing the number of MHA steps per iteration.

**Fig 13:**
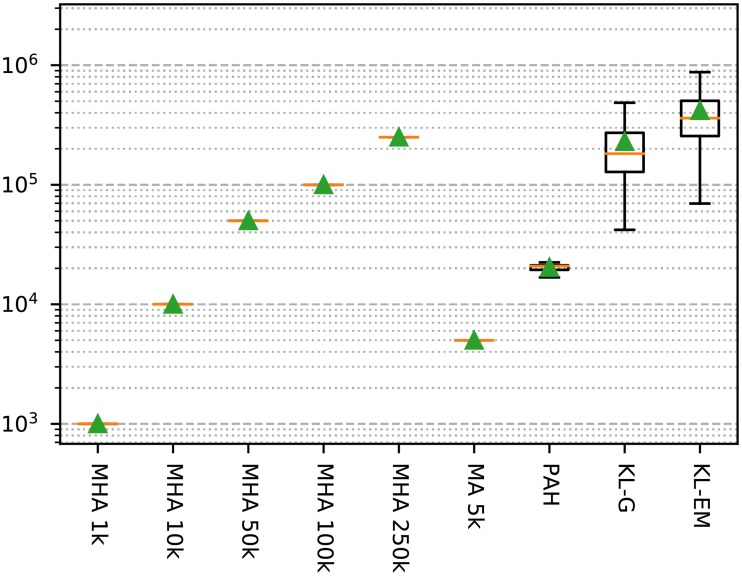
The recorded number of calculated deltas of the studied inference algorithm during all performed runs for Fig 11.

The other side of the performance of the inference algorithm are the needed number of delta calculation, which is shown in Fig 13. Again the variants of the Kernighan-Lin algorithm need the most number calculation, but they still stay in an acceptable scale. Based on these observations, we have selected Metropolis-Hasting algorithm with 250 000 steps as fair balance of result and performance and executed the algorithms with number of blocks from 1 to 30.

**Fig 14:**
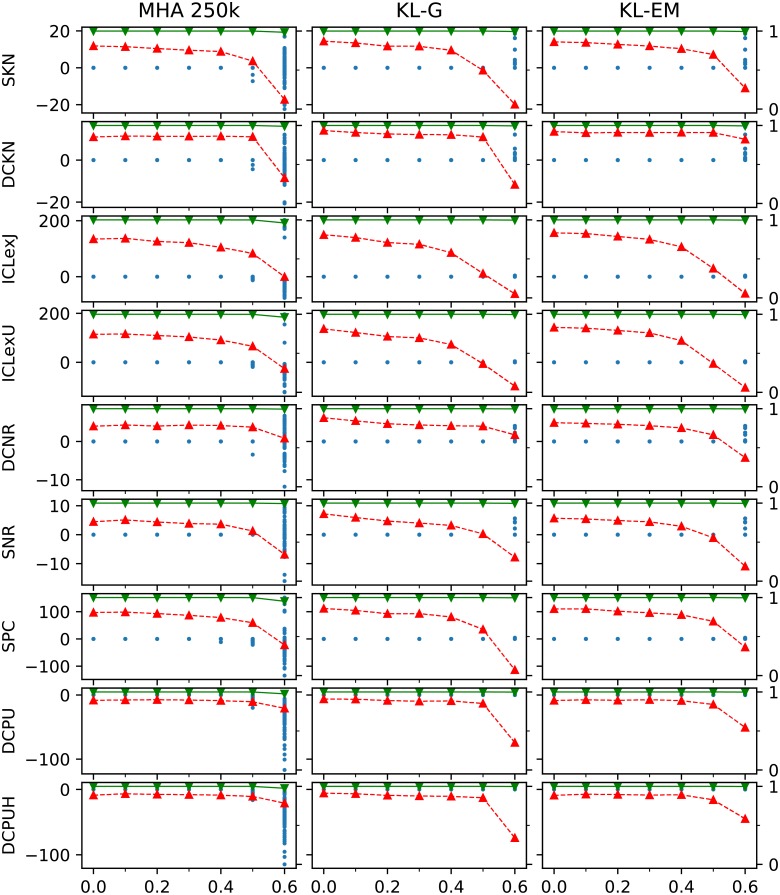
Executing the inference algorithms from the true partition 10 times for 10 network instances for each *μ*_*t*_ = 0, 0.1, …, 0.6 of the LFR benchmark. The Figure is structured in the same way like Fig 6. The resulting difference to the original value (designated with a blue dot ●), the average AMI of all runs of all network with the same parameter (represented with green triangles upside down ▼) and for comparison the average of the results from a random starting partition with the known number of groups (designated with red triangles ▲) are shown. The AMI values of each row are visualized according to the right axis of each row. The difference between the objective function values we calculate with *P*_observed_ − *P*_true_ and is measured according to the left axis in each row.

Like we did for the GN test, we checked the performance of the inference algorithms and the models, if we give the the ideal start from the planted partition. The results in Fig 14 show, that the planted partition is for *μ*_*t*_ ≤ 0.4 very stable. For *μ*_*t*_ = 0.5 we see from the executions of KL-G and KL-EM, that the maximum of all models is still the one of the planted partition. Though the MHA starts to stop at partitions in the near of the planted one, which indicate a lesser concentration on the value of the planted partition. At *μ*_*t*_ = 0.6 all models beside SPC, DCPU and DCPUH tend to have new maximum value at a partition slightly different from the planted partition. Yet, in all cases the resulting partition of the MCMC increases to vary, but all retrieved partitions have AMI values near to 1. As we can see in Fig 14 all models seem to describe the respective networks quite good, but the applied inference algorithm lack to retrieve the true maximum.

**Fig 15:**
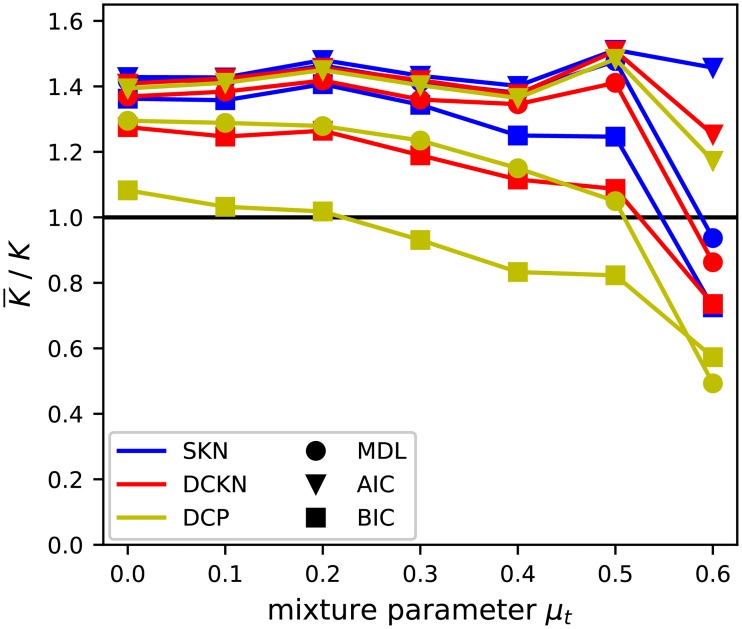
Ratio of average selected number of blocks $\bar{K}$ to average true number of blocks *K* of the classical SBMs. As in Fig 16, the values shown are based on setting of Fig 17.

**Fig 16:**
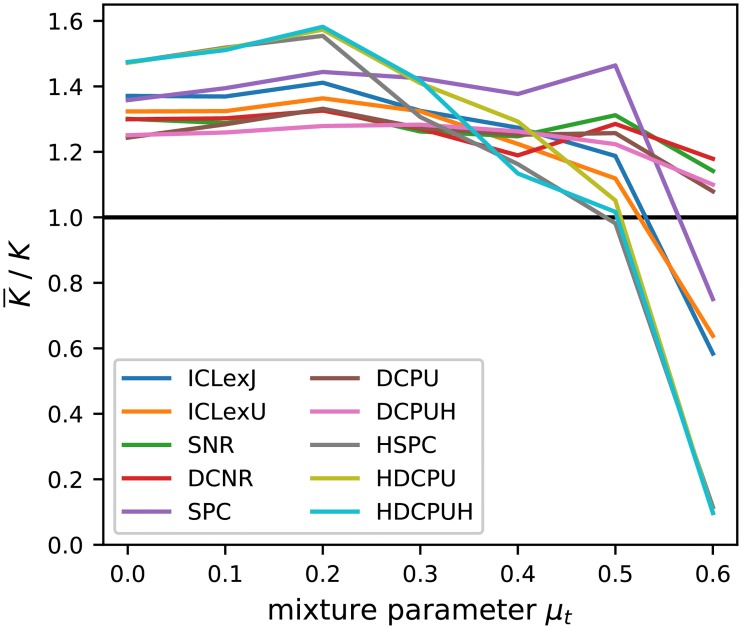
Ratio of average selected number of blocks $\bar{K}$ to average true number of blocks *K* of those SBM variants including model selection. The values shown are based on the same setting as Fig 17.

The results of the classic and the new SBM variants in regard to the relative selected number of blocks in Figs 15 and 16 are closer. For the classic SBM, the AIC always selects too many groups and can only be used to get an upper bound because it never underestimates the number of blocks. The BIC retrieves for all variants the results closest to the true number of blocks. With increased mixture parameter *μ*_*t*_ it shows a tendency of selecting fewer blocks. The MDL delivers results in between those above and has the property to reject too complex structures in favor of a pure random model.

The SBM variants, which includes the size selection, are all prone to overestimate the number of blocks. Most variants are constantly overfitting with the same rate around 30% above the true average value. Only the ICLex variants, SPC and the hierarchical SBMs show a decrease for the most complex case.

**Fig 17:**
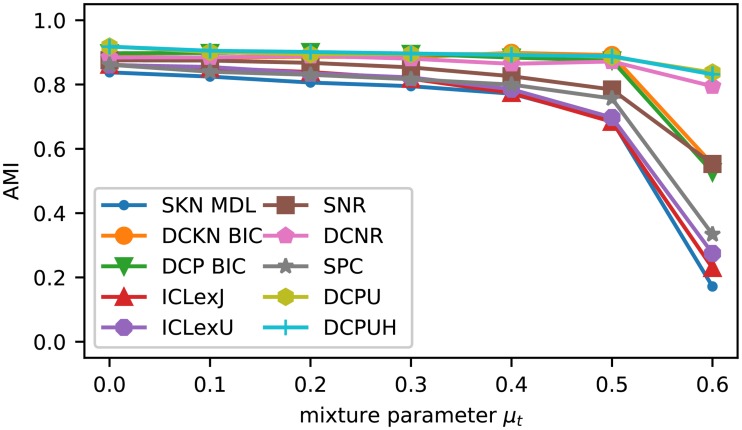
Results of LFR benchmark using model selection. Like before, we used 10 network instances for each *μ*_*t*_. For each network and each SBM variant we executed the MHA 250k 10 times for each *μ*_*t*_ = 0, 0.1, …, 0.6. Then we considered one execution for each network for all number of groups *K* ∈ {1, …, 30} as one unit and executed the model selection based on these results. Therefore, a data point is the average of 100 AMI values resulting from 10 networks and 10 selected partitions. For the classic models only the best model selection according to Table 3 is shown.

The comparison of the results with given number of blocks and with the model selection of Figs 11 and 17 shows an overall decrease of values. But for some variants like SNR, the model selection affects the value more than others like DCPU and DCPUH, which are in top of both cases and their results only show a negligible difference. In general, the difference between those top performing variants and all the others grows with increasing complexity.

For a reduced overview, Table 3 includes the normalized AUMIC for 0 ≤ *μ*_*t*_ ≤ .5 based on the results of the 10 executions of the MHA 250 000 algorithm for each network and each *K* = 1, …, 30. As in the case of the GN test, the results of the hierarchical variants (HSPC, HDCPU, HDCPUH) were created using an adopted version of Peixoto’s agglomerative heuristic with 1000 steps for each MHA execution, which needs in our experiments a comparable amount of delta calculations as the MHA 250 000 algorithm used for the other results. The new degree-corrected SBM variants DCPU and DCPUH of Peixoto achieve the best normalized AUMIC with values of 0.89 and 0.90, which is close to the best value of the GN test. All but one regarded combination of model selection and SBM variant tend to increase their AUMIC with the model selection. This behavior is different from our GN test results and may be an indicator for the tendency of regarded SBM variants to describe LFR networks with different number of groups.

**Table 3:** Normalized AUMIC of the different SBM variants of the LFR benchmark for 0 ≤ *μ*_t_ ≤ 0.5 based on 10 executions of the MHA 250 000 from 10 random partitions for each number of groups *K* = 1, …, 30 and each *μ*_*t*_ = 0, 0.1, …, 0.5.

Variant	True Group Size	With Model Selection	
SKN	0.75	MDL	0.79
		AIC	0.79
		BIC	0.81
DCKN	0.86	MDL	0.86
		AIC	0.85
		BIC	0.89
DCP	0.86	MDL	0.89
		AIC	0.85
		BIC	0.89
ICLexJ	0.70		0.81
ICLexU	0.70		0.82
SNR	0.74		0.85
DCNR	0.78		0.88
SPC	0.77		0.82
DCPU	**0.89**		**0.90**
DCPUH	**0.89**		**0.90**
HSPC	–		0.74
HDCPU	–		0.76
HDCPUH	–		0.74

### Real world networks

The computer generated benchmarks have the advantage that the complexity can be tuned and that arbitrary number of networks with known group structure are available. But with regard to some characteristics, like clustering coefficient or type of community structure, those models still differ from their real world counterparts. Therefore, we have selected some real world networks with available metadata about the division of nodes into groups. Such information is sometimes called ground truth, but some authors argue such information described different structural properties than the topological groups aimed by currently available clustering algorithms [16]. Moreover, the metadata could in theory partition the given network into any partition and thus the limitations of the no free lunch theorem [14] apply for this part of our analysis. At this point we are interested in the degree of similarity between inferred results of SBM variants and the given metadata.

We have decided to compare the presented SBM variants on three real networks with comparable basic properties of the benchmarks mentioned above. As first network, we selected the network of American college football, created by adding an edge between competing teams for each match of the season 2000 [11]. The original data in [11] had some issues like the assignments of teams from 2001 and not the correct one from the year 2000. Therefore, we use a corrected version of Evans [95]. The next complex network is a co-purchasing network of political books around the time of the American presidential election 2004 [96]. The last data set is a network of links between political blogs [97]. The metadata available for these networks consist of the conferences, the categorization into the political directions conservative, liberal for the political books, and conservative, liberal, and neutral for the blogs. This selection is based on the selection of traditional cases by Hric et al. [16] and for comparability we followed them by reducing the network to the largest weakly connected component and removing any present multiple edges. The political blogs’ network is a directed network and to apply the SBM variants in the presented form, we transformed the network into an undirected network. Since our implementation includes their extension to the directed cases as well, we tested both possibilities.

**Fig 18:**
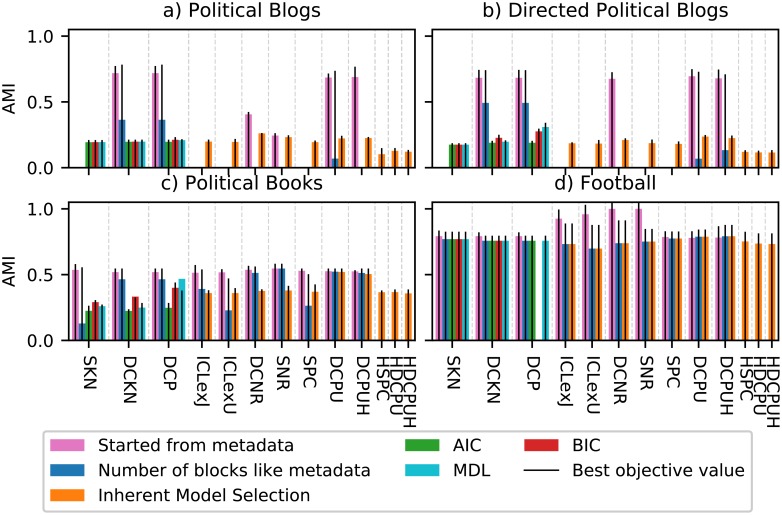
Results of inference of real networks. Each model beside the hierarchical ones (HSPC, HSDCPU, HDCPUH) was executed 10 times with MHA with 250 000 steps from the partition given by the metadata and for each group size between 1 and 15 (respectively 25 for the football network) from 10 random starting points. For these models the figure includes results of the start from the metadata and for partitions with the same number of groups like the metadata. The classical SBM also contain the results for the three presented model selection AIC, BIC and MDL. Whereas, the models, which include the model selection step, show instead the results of their model selection. The hierarchical SBM variants only show the results of 10 executions with algorithm for the hierarchical models. The bars represent the average of the 10 independent runs (for the one with model selection one execution per group size) and the solid line in the middle of each bar represents the AMI of the partition with the best objective value. Bars not visible relate to results near to zero and the dashed gray line with its white space was added to separate the models.

Like in the benchmarks, we have executed the greedy (KL-G) and the EM variant (KL-EM) of the Kernighan-Lin algorithm to get an idea of the needed number of steps for the Metropolis-Hastings algorithm. Then, we applied the Metropolis-Hastings algorithms 10 times for each block size from 1 to 15. As before, Fig 18 contains the results of all SBM variants with the numbers of groups like the metadata and with model selection for each network.

The similarity between the metadata and the inferred SBM partitions of the political blogs network is quite low with AMI values around 0.2. Even starting from the metadata results in moderate AMI values. A detailed analysis reveals the categorization of the nodes according to their degree instead of other topological structures. Fig 2 shows the inferred partitions into two groups with the highest observed likelihood for the SKN and the DCKN SBM variant. Furthermore, we observe this phenomenon not for standard SBM variants only, but for some degree corrected variants as well. Including the direction information, does not significantly change the agreement between metadata and inferred partitions. In this case, the information about the direction of the links seem to add no further information to the applied SBM variants.

For the political books, we get slightly better results than for the political blogs, but still most SBM variants deliver poor agreement. Yet, this time there is no strong division by node degree.

As we can see, all variants deliver good results for the football network. For some of the tested SBM variants the partition given by the metadata is a stable local optima and the selected partitions from random starting points still strongly agree with the metadata. To our surprise, the same number of groups like the metadata is retrieved by many variants and this constantly in every execution. Yet, most algorithms fail to cluster the independent teams in the same way like the metadata.

During the execution of the SBM variants on the real networks, we have encountered the division according to the node degrees for some non-degree-corrected and some degree-corrected SBM variants. This confirms the finding of Karrer and Newman on bigger networks [32] and unlike their findings for the Zachary’s karate club network the local optima retrieved in our study are stable and not metastable, i.e. the best optima we were able to retrieve. Yet, we only saw this phenomenon for partitions with exactly two clusters.

## Discussion & conclusion

We presented the different approaches to develop a Stochastic Block Model, which are influenced by various disciplines. Moreover, we showed how this diversity led to plurality of formulation and variants of the basic idea of SBM. With our comparison of the complete process from the rationale behind the SBM variants, over different approaches to deal with the model selection, to inference algorithms for the maximization of the resulting objective function, we highlighted the advantages and disadvantages of the existing solutions.

In our review of SBM variants, we presented different formulation based on the same principle of structural equivalence. Some authors used a rather continuous approach, which often in a natural way include extension to (continuous) overlapping groups. Others like Peixoto stated the SBM using combinatorics, which enabled them to reduce the bias of priors by building a hierarchy of hyperpriors.

In our analysis of the inference methods we have seen the interplay of performance, efficiency and design of the different approaches. In our tests Peixoto’s agglomerative heuristic was good in relation to performed delta calculations, but had a great overhead caused by the bottom-up approach. Furthermore, its two parameters should be chosen in respect for the size and properties of the observed network. For small cases the Kernighan-Lin algorithm is the best choice with regard to its continuously good results, that comes at the expense of largest theoretical and observed run time. Using trial runs of the faster variants of the Kernighan-Lin algorithm, we overcome the weakness of selecting the number of steps for the Metropolis-Hastings algorithm. Based on the design of our study, we focused on the interplay of the SBM variant and the inference method, and had to exclude some promising approaches such as belief propagation from our analysis.

Like every comparison of community detection algorithms, our work has some limitations. We evaluated the performance of the SBM variants and inference algorithm on an extended version of the GN test and the more general LFR benchmark and varied the mixture of the blocks. However, the performance of the algorithms is influenced at least by the factors: degree distribution, average degree, distribution of community sizes, connectivity pattern, and size of the network. The modification of one or more of these parameters for the creation of artificial networks is therefore likely to lead to different results.

Another point raised by the work of Peel et al. [14], is the inherited challenge of our comparison based on evaluating the quality of retrieving a planted partition which was used to generate the network given to the algorithm. Ghasemian et al. [15] proposed as solution for this the usage of a large set of real networks instead of artificial ones and a combination of the two measures link prediction and link description. Yet, the calculation of these measures introduce an additional layer by requiring the algorithms to rank all edges. For this layer, we see again multiple choices like the changes of the objective function induced by the new edge or the probability given by the generative process with or without taking the possible new edge into account. Additionally, the link prediction is unclear for all algorithms that do not directly maximize an objective function or are subject to a generative model, such as spectral algorithms. In addition, at least the link prediction can be improved by taking not only a single but multiple partitions into account [6], which probably influences the performances. Therefore, comparisons based on artificial networks are still needed before raising the complexity with an additional choice.

During our analysis, we have identified some characteristics of the studied SBM variants. First, the selection of the correct number of blocks is still challenging. Maybe the area of the true value could be bounded from below and above in addition to retrieving the likeliest value, which would be a great advantage in real world applications. This information could be combined with the property of rejecting weak structure or returning significance value of the result. The latter is itself a desirable feature, but having the no free lunch theorem [14] in mind, this would reveal the subset of networks for which the variant is designed. Next, as one result of our extension of the GN test, the compared SBM variants seem to have an inherited and different strong tendency to prefer assortative groups instead of disassortative ones. This property should be made more explicit or at best all implicit bias could be overcome. At last and in accordance with those results of [16], we saw a discrepancy between known metadata and the retrieved topological based blocks. Some SBM variants rejected any present structure by dividing the nodes according to their degree, which could indicate that, from a topological view, in those cases no structure is detectable.

Finally, by supplying the code to test all presented SBM variants and inference methods on own data or with new modification, we aim to contribute to the identification of the above mentioned characteristics.
